# AuAg–Carbon-Based Quantum Dot Nanocomposites to Reduce Andrographolide’s Hydrophobicity and Drug Internalization Tracking in PC-3 Cells

**DOI:** 10.3390/nano16070396

**Published:** 2026-03-25

**Authors:** Nataniel Medina-Berríos, Alondra Veloz-Bonilla, Sebastián C. Díaz Vélez, Mariana T. Torres Mulero, Kim Kisslinger, Alejandro O. Rivera-Torres, Gerardo Morell, Magaly Martínez-Ferrer, Brad R. Weiner

**Affiliations:** 1 Department of Chemistry, University of Puerto Rico, Rio Piedras Campus, San Juan, PR 00925, USA; sebastian.diaz8@upr.edu; 2 Molecular Sciences Research Center, University of Puerto Rico, San Juan, PR 00926, USA; alondra.veloz@upr.edu (A.V.-B.); mariana.torres9@upr.edu (M.T.T.M.);; 3 Department of Biology, University of Puerto Rico, Rio Piedras Campus, San Juan, PR 00925, USA; alejandro.rivera17@upr.edu; 4 Brookhaven National Lab, Upton, NY 11973, USA; 5 Division of Cancer Biology, University of Puerto Rico Comprehensive Cancer Center, San Juan, PR 00927, USA; magaly.martinez1@upr.edu; 6 Department of Physics, University of Puerto Rico, Rio Piedras Campus, San Juan, PR 00925, USA; 7 Department of Pharmaceutical Sciences, School of Pharmacy, Medical Sciences Campus, University of Puerto Rico, San Juan, PR 00936, USA

**Keywords:** nanoparticle, nanocomposite, carbon-based quantum dots, gold-silver alloy, drug delivery, hydrophobic drugs, prostate cancer

## Abstract

Hydrophobicity has limited the efficiency of many drugs. To improve this, gold–silver alloy nanocomposites covered with carbon-based quantum dots were synthesized as a platform to reduce the drugs’ hydrophobicity. Using the hydrophobic drug Andrographolide as a model, it was demonstrated that these nanocomposites can decrease Andrographolide’s hydrophobicity (Log P from 2.632 to 0.56) without encapsulating the drug. Entry within prostate cancer (PC-3) cells and in vitro localization of the nanocomposites and Andrographolide was observed qualitatively via confocal microscopy and their identity confirmed by SERS inside the PC-3 cells. MTS assays demonstrated the carbon-based quantum dot layer covering the metal core of the nanocomposites stabilizes the oxidation rate of the nanocomposite’s core metals. This was observed by a decrease in cytotoxicity in PC-3 cells when compared to other gold or silver nanosystems for similar timeframes published in the literature.

## 1. Introduction

Carbon-based quantum dot (CBQD)-covered metal nanocomposites can enhance the properties of the nanoparticles and help overcome the drawbacks of the core material [1]. In the case of nanoparticles with noble metal (Au, Ag) cores, a covering layer helps prevent agglomeration and surface oxidation [2]. In the case of many carbon nanomaterials, serving as a covering layer may also impart other intrinsic properties, i.e., fluorescence. Graphene [3], graphene oxide [4], and CBQDs [1,2,5] have been successfully used as coatings for gold, silver and gold–silver nanoparticles of different geometries, enhancing the effectiveness of their intended applications and lessening counterproductive features. In this work, a coating of CBQDs on a gold–silver alloy core is used as a multi-functional platform to help solubilize hydrophobic drugs, as well as provide drug delivery tracking via its intrinsic fluorescence. This type of nanocomposite was chosen among different existing nanoparticles as a possible complement to clinically established liposomes, niosomes, cubosomes, transfersomes and ivosomes who have been reported to have rapid clearance, limited drug loading capacity, drug leakage and loading versatility, among other issues [6]. The stability and biocompatibility afforded by the CBQDs’ coating combined with the ability of gold–silver alloys to curb the cytotoxicity that silver nanoparticles exhibit, increasing the plasmonic activity compared with pure gold nanoparticles, provide promising capabilities for a powerful multifunctional drug delivery agent [7].

More than 40% of drugs in clinical use are hydrophobic and ≥90% of new drug candidates may be described as either Class II (high permeability, low solubility) or Class IV (low permeability, low solubility) in the Biopharmaceutics Classification System [8]. Andrographolide (ADG) is a bicyclic diterpene-g-lactone, a natural product with anti-cancer properties found in the plant *Andrographis paniculata*, which has been demonstrated to possess anti-prostate cancer properties [9]. It has a large 1-octanol/water partition coefficient (Log P) of 2.632 ± 0.135 [10] and a Log D at pH 7.4 of 1.4 [11], which affects parameters such as absorption, distribution, permeability and routes of drug clearance [12]. ADG was selected as the hydrophobic target and PC-3 cells for the in vitro cancer cell model to assess drug internalization tracking.

Nanoparticle uptake mechanisms for cell internalization are known to involve a variety of pathways, such as phagocytosis, micropinocytosis and endocytosis [13], where each of them has its unique implications for drug delivery. These mechanisms, along with transcellular and paracellular transport, determine how effectively a drug carrier can penetrate biological barriers. The uptake route of a nanoparticle depends on its physical and chemical properties, such as size, shape, surface charge and hydrophobicity. The use of gold–silver alloy nanocomposites covered with CBQD (AuAgCBQD) nanoparticles allows manipulation of these properties to facilitate cellular and trans-barrier internalization via endocytosis. Specific pathways, such as Clathrin-mediated or Caveolae-mediated endocytosis (CME), can be influenced by the nanoparticle’s drug coating and the interactions between receptor and ligand, but the absence of functional Caveolae in the surface of PC-3 cells suggests that CME is the predominant mechanism for these nanoparticles [14,15].

Upon cellular entry, AuAgCBQD nanoparticles—whether drug-coupled or not—can be visualized using confocal microscopy. This advanced imaging technique enables the observation of nanoparticles after they have penetrated the cell, validating their intracellular localization. The application of confocal microscopy to AuAgCBQD nanoparticles provides insights into their endocytic pathways and the fate of the associated drug delivery systems. The distinctive fluorescence of CBQD-coated metal nanocomposites is instrumental in monitoring cellular and trans-barrier internalization, thereby enhancing the tracking of therapeutic delivery within the cellular environment.

To adequately identify and monitor the process of uptake, distribution and release of drugs within targeted cells, certain methods of intracellular drug detection have been employed. Examples of these methods include fluorescence imaging [16], radioisotope labeling [17], confocal microscopy [18] and Raman spectroscopy [19]. For this study, we focused on confocal microscopy and Raman spectroscopy. Adequate drug delivery detection and evaluation is important for identifying mechanisms of uptake, intracellular drug distribution, efficacy of delivery method, drug toxicity and the determination of adequate dosing. Confocal microscopy utilizes a laser beam to excite the sample, and the fluorescence is subsequently collected. The technique is suitable for imaging of fixed as well as live specimens [20]. Raman spectroscopy, a technique based on the inelastic scattering of incident radiation coupled to the normal vibrational modes of the targeted molecules, allows selective, but insensitive, detection. Surface-enhanced Raman spectroscopy (SERS) improves the intensity of the Raman bands by using metals as a substrate [21], which allows for the detection of molecules at low concentrations. SERS, as opposed to other Raman spectroscopy methods, provides better sensitivity, monitoring and identifying mixture components, making single molecule detection possible [22]. In this work, we synthesized gold–silver alloy nanocomposites covered with CBQDs, derivatized with doped-CBQDs (D-CBQDs) to modulate surface properties, for solubilizing the hydrophobic drug Andrographolide and monitoring its delivery to PC-3 cells via confocal microscopy and SERS.

## 2. Materials and Methods

Materials were purchased from Sigma Aldrich (Saint Louis, MO, USA), Fisher Scientific (Cayey, PR, USA) and Ted Pella (Redding, CA, USA). Cell lines PC-3 and RWPE-1 were obtained from ATCC (Manassas, VA, USA). All the solutions were prepared using deionized water by the Aries Filter Works Gemini model GMS-105 with GMA-UV, phosphate-buffered saline (PBS; Sigma Aldrich) and ethanol. UV-Vis measurements were recorded on a Thermo Fisher Scientific, Genesys 10S UV-Vis spectrophotometer. Fluorescence spectra were obtained with a Shimadzu RF-6000 Spectro fluorophotometer from Shimadzu Corporation (Columbia, MD, USA). Absorbances for cell viability tests were done using a TECAN infinite M200PRO microplate reader. Raman spectra were obtained from a Thermo Fisher Scientific DXR Raman Microscope with a DXR 532 nm filter and OMNIC (Version 9) as the data acquisition software. Sputtering was done using a Pelco SC-7 Auto Sputter Coater with a 57 × 0.1 mm 99.99% Ag target. NMR characterizations were performed on a Bruker Ascend Aeon 500 using deuterium oxide (D_2_O) as solvent and Bruker TopSpin 3.5 software for data acquisition. The solvent signals at 4.80 and 4.81 ppm were used as internal standards for protons. To study the morphology and dimensions of nanocomposites, X-ray microanalysis was recorded with a JEOL JSM-6480LV scanning electron microscope from JEOL USA (Peabody, MA, USA) with an Evenhart Thomley secondary electron imaging (SEI) detector and an energy dispersive X-ray analysis (EDS) Genesis 2000 detector and FEI TALOS 200× high-resolution scanning/transmission electron microscope (TEM). Size distribution was realized using ImageJ software (Version 1.54p) [23]. Confocal microscopy was done withNikon Ti Microscope from Nikon USA (Melville, NY, USA) with a S Fluor 40× Oil DIC H N2 objective. Confocal microscopy channels, Alexa Fluor 488 (Exc 487.5 nm, Em 525.0 nm) and GFAP CY3 (Exc 561.5 nm, Em 595.0 nm), were used with a Laser Scan Confocal GaAsP modality and TD (Exc N/A, Em N/A) with a Laser Scan Confocal TD modality. Capturing was done with a Z step of 0.3 µm with a count of twenty-five.

### 2.1. Synthesis of AuAgCBQDs and AuAgDCBQDs

CBQDs, S-CBQDs and N-CBQDs were synthesized as described in a previous report [24]. CBQDs and D-CBQDs were used as reducing agents for a 1:1 proportion mixture of 150 µM HAuCl_4_ and 150 µM AgNO_3_ aqueous solutions. UV-Vis was used to adjust the concentration of CBQDs and D-CBQDs to one with just enough reducing activity [25]. Complete reduction in metal ions was confirmed by SEM-EDS, i.e., no metal ions were observed. Since SEM-EDS is a surface technique, no metal ions observed means that the CBQDs and D-CQBDs reduced all the metal ions and had covered the surface of the metal core completely. Trials were done increasing the concentration of CBQDs until SEM-EDS showed no presence of free Au and Ag ions.

### 2.2. Drug Loading of Andrographolide

Dry AuAgCBQD, AuAgNCBQD or AuAgSCBQD were dissolved in water and mixed with an ethanolic solution of Andrographolide in a 1:1 volume ratio. The mixture was sonicated at 50 °C for 30 min and then rotoevaporated.

### 2.3. Partition Coefficient (Log P) Determination

The sessile drop method was not selected due to the limitations of this method, such as variable results depending on surface factors, like roughness and heterogeneity, environmental sensitivity and contamination [26]. In addition, due to the complexity of biological surfaces, the contact angle measurements fall short of being able to assert the hydrophobicity or hydrophilicity of a formulation. On porous or porous-like biological surfaces, the contact angle decreases with time as the drop is absorbed, making it difficult to define a single, stable “equilibrium” contact angle [27,28]. For these reasons, we believe the combination of the Log P value and the NMR chemical information to be a more accurate method to determine the changes in hydrophobicity in this context [29]. Dry samples were dissolved in D_2_O and added to an NMR tube, and the ^1^H-NMR spectrum of each sample was taken. Next, 1-octanol was added to match the amount of D_2_O in each sample, vigorously mixed, and left to reach equilibrium for 1 h. The D_2_O phase was then remeasured by NMR. The peaks were integrated and normalized to their respective D_2_O peak before applying the formula:

(1) $$\mathrm{Log}(K_{ow})=\mathrm{Log}(\frac{{\mathrm{RI}}_{w}^{\mathrm{init}}-{\mathrm{RI}}_{w}^{\mathrm{equil}}}{{\mathrm{RI}}_{w}^{\mathrm{equil}}})$$

where *RI_W_^init^* is the relative NMR signal integration of the materials in D_2_O before equilibration with 1-octanol and *RI_W_^equil^* is the relative NMR signal integration of the materials in the aqueous phase after equilibration. The obtained *K_ow_* is then utilized as the partition constant and its logarithmic value (Log *K_ow_*) is considered as the obtained Log P [30].

### 2.4. Preparation of Ag-Sputtered Hydrophilic PVDF Membranes as SERS Substrates

A commercially bought Durapore filter (Fisher Scientific; diam. 25 mm) of hydrophilic PVDF membrane with 0.1 µm pore size was sputtered with a silver target at a rate of 1 nm of Ag/second under argon gas. The hydrophilic PVDF membranes sputtered with 80 nm of silver were prepared using focused ion beam milling (FIB) and characterized by HR-TEM and in situ EDS to confirm the exact thickness of the silver.

### 2.5. Cell Culture

PC-3 cells for confocal microscopy were cultured using F-12K medium supplemented with 10% fetal bovine serum (FBS) and 5% antibiotic–antimycotic solution. For cell viability, PC-3 cells were cultured using RPMI 1640 medium supplemented with 10% FBS and 5% antibiotic–antimycotic solution. Cells were grown to a 70% confluency for all experiments. Cells were seeded in their respective microplates for all applications at a confluence of 7 × 10^4^ cells/mL and incubated until the next day to allow them to adhere to the surface of the plate(s). For MTS assays, RWPE-1 cells were grown using Keratinocyte Serum Free Medium (K-SFM) supplemented with 0.05 mg/mL bovine pituitary extract (BPE) and 5 ng/mL human recombinant epidermal growth factor (EGF). RWPE-1 cells were grown at 37 °C and 5% CO_2_ atmosphere. Cells were grown to 70% confluency and incubated for 48 h with media containing the nanomaterials. MTS assays were performed following the manufacturer’s instructions.

For confocal microscopy, attached and confluent cells were incubated with an excess of nanomaterial to observe entry and accumulation of the nanoparticles within the cells for 1 h, 2 h and 4 h time points. At each time point, media with nanomaterial was discarded, cells were washed three times with Dulbecco’s PBS, fixed with freshly prepared formaldehyde (4% in PBS) for 10 min, washed three times with Dulbecco’s PBS and mounted in media for confocal imaging (50% glycerol and 50% Dulbecco’s PBS 1X).

For SERS detection, cells were grown in 96-well plate(s) and then incubated with medium containing the nanomaterial for 2 h. The cells were then washed three times with Dulbecco’s PBS, detached either using Tryp-LE and filtered using a vacuum filter unit with a Ag 80 nm PVDFh membrane or using a scraper and depositing the cells onto the Ag 80 nm PVDFh membrane. Both methods of detaching the cells provided similar quality in the observed Raman spectra. Raman microscopy was done using the Ag 80 nm PVDFh membrane as a substrate. The laser power was adjusted to 3 mW. Intact cells with no cell or medium debris nearby were selected to measure the SERS spectra. The obtained spectra were processed using OriginPro 2017 software.

Data processing and ANOVA tests for the MTS assays were done on Microsoft Excel for Microsoft 365 MSO (Version 2312 Build 16.0.17126.20132) 64-bit. Trend fitting was performed with the OriginPro 2017 software using the Boltzmann function with the Orthogonal Distance Regression iteration algorithm [31]. IC_50_ values were calculated by solving for the given equation resulting from the trendline when y = 50 (“y” being normalized percentage of inhibition).

## 3. Results and Discussion

### 3.1. Transmission Electron Microscopy (TEM) and EDS

**Figure 1:**
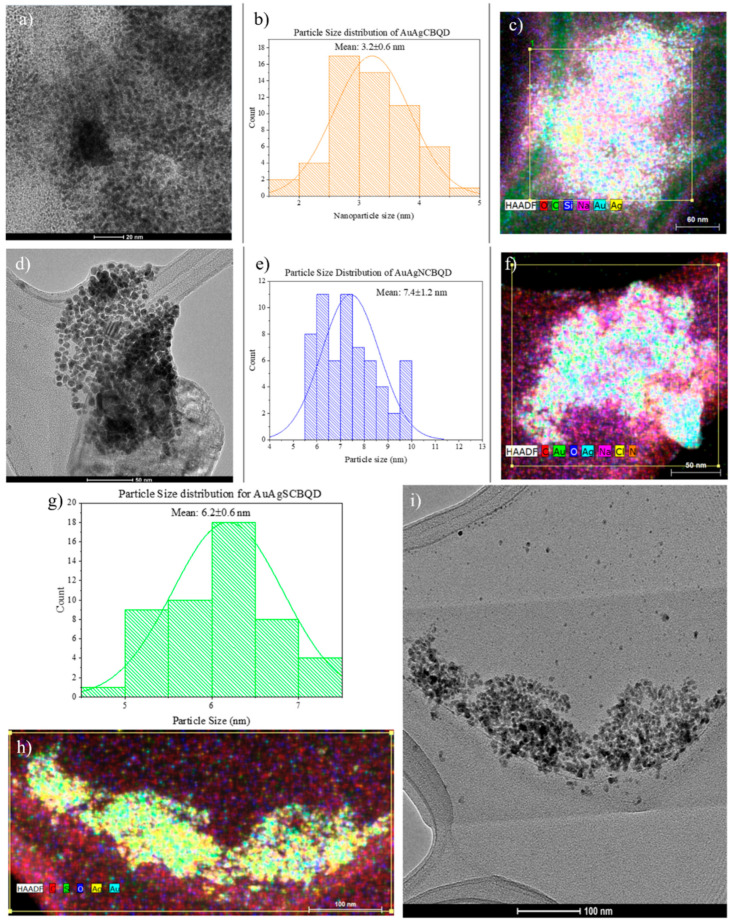
(**a**) TEM image of AuAgCBQD, (**b**) particle size distribution of AuAgCBQD, (**c**) EDS map of AuAgCBQD; (**d**) TEM image of AuAgNCBQD, (**e**) particle size distribution of AuAgNCBQD, (**f**) EDS map of AuAgNCBQD; (**g**) particle size distribution of AuAgSCBQD, (**h**) EDS map of AuAgSCBQD, (**i**) TEM image of AuAgSCBQD. Uncertainty of size distribution corresponds to statistical error when measuring nanoparticle size in Image-J [23].

Figure 1 shows the size distribution, morphology and elemental analysis of the synthesized gold–silver alloy nanocomposites covered with the CBQDs and D-CBQDs obtained by TEM-EDS. Among the three composites, AuAgCBQDs were the smallest (3.2 ± 0.6 nm), while AuAgNCBQDs were the largest (7.4 ± 1.2 nm). AuAgSCBQDs had an intermediate size of 6.2 ± 0.6 nm. All particles displayed an irregular quasi-spherical morphology [32].

Elemental analysis by EDS showed the proportion of gold and silver that was reduced to form the nanocomposites. The gold and silver content varied depending on which CBQD/D-CBQD was used as a reducing agent even when all mixtures started as a 1:1 ratio of Au and Ag (see Table 1). This difference results because doping changes the electronic structure and surface chemistry of the CBQDs, which directly affects their reducing potential toward the metal ions [33]. In all three nanocomposites, a higher content of silver was observed. Doping of the D-CBQDs was observed in the EDS map of the nanocomposites, confirming the presence of the CBQDs’ layer.

### 3.2. Nuclear Magnetic Resonance (NMR) Studies

**Figure 2:**
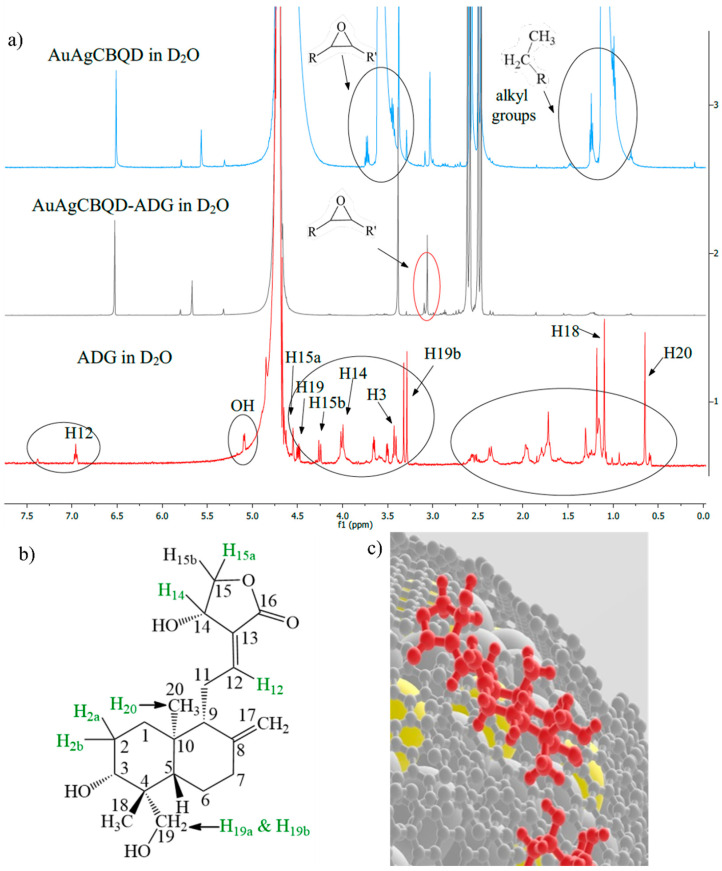
(**a**) ^1^H-NMR spectroscopy comparing AuAgCBQD alone, AuAgCBQD with the drug (AuAgCBQD-ADG), and the drug alone (ADG). (**b**) Andrographolide’s molecular structure with assigned protons. (**c**) 3D representation of ADG’s association to AuAgCBQD’s surface. Silver small spheres are the CBQDs, silver big spheres are silver atoms, yellow spheres are gold atoms and in red is ADG.

^1^H-NMR spectra (see Figure 2) showed that the AuAgCBQDs and AuAgDCBQDs have the functional groups of their respective CBQDs and D-CBQDs present. When loading Andrographolide onto the nanoparticles, differences were observed in the spectra. ADG, being relatively flat structurally, exhibits interesting interactions when coupled onto the surface of an irregular quasi-spherical shaped nanoparticle. This interaction leads to specific NMR spectral changes, reflected in changes in the chemical shifts (ppm values) of certain peaks. These changes are particularly evident with the AuAgNCBQD nanoparticle, characterized by increased surface polarity, and in the case of AuAgSCBQD with increased doping atom polarizability, which can alter the electronic environment around the ADG’s protons. Figure 2b shows the molecular structure of ADG with the protons numerically labeled for reference in the discussion below. As the NMR showed no lone drug signals, it is assumed that it is completely associated with the nanoparticle to the extent of the resolution of the instrument.

In a comparative NMR spectroscopic analysis, the AuAgCBOD nanocomposite was observed to undergo significant structural changes when modified from AuAgCBOD-ADG. The disappearance of proton signals at 6.98 ppm, 3.56 ppm, and 3.38 ppm in the modified compound indicates the alteration of specific functional groups. The 6.98 ppm triplet, assigned to the H_12_ proton in ADG, is no longer present, suggesting that a modification occurred at that site. Similarly, the signals at 3.56 ppm and 3.38 ppm, previously attributed to CBQD epoxy groups and a CBQD proton adjacent to an alcohol group, respectively, have vanished, suggesting a reaction involving these groups. The emergence of a new signal at 3.06 ppm in the AuAgCBOD-ADG spectrum is particularly noteworthy. This new signal is indicative of an epoxide functional group, which may be a result of the interactions between AuAgCBQD and ADG.

Based on the ^1^H-NMR analysis of AuAgCBQD, the derivatives, AuAgNCBQD and AuAgSCBQD, were characterized and the chemical shift changes observed in the NMR spectrum of ADG coupled with the nanoparticles were summarized. In the case of coupling of ADG with AuAgNCBQD, the triplet at 6.96 ppm corresponding to ADG’s H_12_ protons remains unchanged, suggesting that these protons’ chemical environment is not significantly perturbed by the nanoparticle. However, several signals, including the drug’s singlet at 4.55 ppm for H_15a_, the multiplet at 4.49 ppm for H_19_, and the doublet at 4.01 ppm for H_14_, disappear after coupling. This indicates that the protons in these positions are affected by coupling, due to interactions with the nanoparticle that alter their environment. Additionally, the doublet at 0.59 ppm associated with H_20_ also disappears, hinting at an involvement of these protons in the coupling process or a change in their spatial arrangement.

In contrast, when ADG is coupled with AuAgSCBQD, a new singlet emerges at 5.83 ppm, which is not present in the free ADG. This new signal could be attributed to protons near a double bond that have been affected by the coupling, reflecting a change in the electronic structure around that bond. Moreover, the triplet at 1.79 ppm, associated with ADG’s H_2_ protons, is not observed in the coupled form, indicating a modification in the environment surrounding these protons. The doublet at 0.59 ppm corresponding to H_20_ protons is again absent, consistent with the observations made with AuAgNCBQD, suggesting a similar alteration of the Andrographolide’s structure, when coupled with either type of nanoparticle. These observations indicate a non-uniform association of the drug with the nanoparticle surface, leading to fragments of the drug that do not interact with the nanoparticle.

The NMR spectral data presents that the drug, being structurally flat, exhibits distinct interactions when coupled on the surface of the irregular quasi-spherical nitrogen-doped nanocomposite. This interaction leads to specific NMR spectral changes, reflected as deviations in the chemical shifts (ppm values) of certain peaks and disappearance of others due to Nuclear Overhauser Effect (NOE) [34]. These changes are particularly evident with the AuAgNCBQDs, characterized by the increased surface polarity of the N doping atom [35] and reduced polarizability and smaller atomic radius when compared to the S atom [36]. This in turn, can alter the electronic environment around the ADG’s protons and, consequently, alter the chemical shifts observed in the NMR spectrum. Protons in more polar regions may experience different shifts compared to those in less polar regions, resulting in the disappearance of some peaks or the emergence of new ones. Such characteristics indicate a non-uniform association of the drug with the nanoparticle surface, leading to discernible areas where the drug does not interact with the nanoparticle. This lack of uniformity in association is likely due to the disparity in polarity and the incongruent shapes of the flat drug molecule and the irregular quasi-spherical surface of the nanoparticle. The selective nature of this association is significant, as it may impact the stability of the drug–nanoparticle conjugate and affect the drug’s bioavailability and efficacy in biological systems [37]. All NMR spectra are available in the Supporting Information (Figures S4–S10).

### 3.3. Nuclear Magnetic Resonance Log P Studies

**Figure 3:**
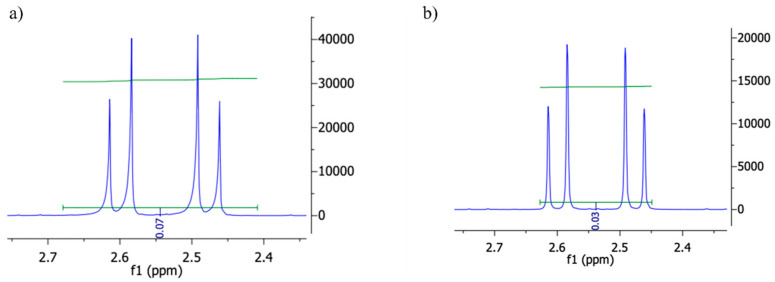
(**a**) Close up of an AuAgCBQDs’ ^1^H-NMR’s peak before addition of 1-octanol. (**b**) Close up of AuAgCBQDs’ ^1^H-NMR’s peak after addition of 1-octanol, mixing and equilibrium of 1 h [30].

When observing the scale of each spectrum in Figure 3, it is observable how the intensity of the peak drops after the addition of 1-octanol (followed by mixing and 1 h equilibrium) due to a fraction of the sample in D_2_O migrating to the 1-octanol phase.

These measurements in Table 2 reflect the nanocomposite-associated drug partitioning. ADG’s Log P (2.632 ± 0.135) [10] was lowered when coupled with the AuAgCBQDs, AuAgNCBQDs and AuAgSCBQDs to around 0.6. In contrast to the Log P of the lone nanoparticles and our previous work with lone CBQDs and ADG [24], the homogenous decrease in hydrophobicity of the drug when coupled to the quasi-spherical nanocomposites may indicate that the decrease in hydrophobicity is more dependent on steric factors than surface polarity, while surface polarity provides the chemical basis for coupling [38,39].

**Table 2:** Log P results by NMR method. σ = standard deviation.

Sample	$\bar{x}$	σ
Andrographolide [24]	1.106	0.02
AuAgCBQD	0.684518	0.05
AuAgCBQD-ADG	0.561179	0.07
AuAgSCBQD	0.829365	0.05
AuAgSCBQD-ADG	0.607821	0.09
AuAgNCBQD	0.823827	0.03
AuAgNCBQD-ADG	0.60637	0.09

### 3.4. Fluorescence Spectroscopy

Excitation and emission spectra of the CBQDs and D-CBQDs that serve as a cover for the nanocomposites were measured at different physiological pHs present in cancer tissue environments to anticipate any possible wavelength shifts. No significant shifts in excitation or emission wavelengths were found at the tested physiological pHs present in cancer (see Figures S11 and S12) [40]. This showed that the nanoparticle’s fluorescence spectra would not be pH sensitive at the ranges present inside and around non-metastatic tumor PC-3 cells [41]. This was important to determine suitable parameters for laser scanning confocal microscopy. Based on these results, the wavelengths chosen for confocal microscopy imaging were an excitation wavelength of 487.5 nm, detecting emission of 525 nm, and an excitation wavelength of 561.5, detecting emission of 595 nm.

### 3.5. Confocal Microscopy

**Figure 4:**
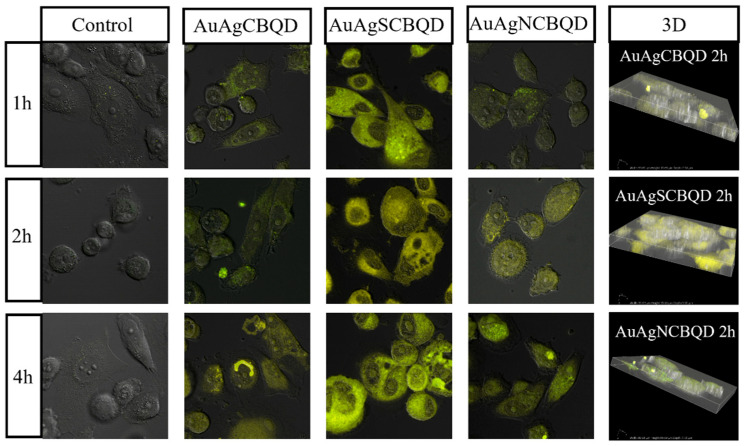
Confocal microscopy of AuAgCBQD, AuAgSCBQD and AuAgNCBQD incubated with PC-3 cells at different timeframes. Deconvolution was done via NIS-Elements Offline Deconvolution software (Version 5.41.00). 3D images confirm material internalization.

The confocal microscopy images shown in Figure 4 provide visual evidence of nanoparticle entry into PC-3 cells. The observed bright yellow fluorescence within the cells in the images labeled with nanoparticle names, as opposed to the control images, is a clear indicator of nanoparticle uptake. For instance, the AuAgCBQD nanoparticles demonstrate increasing fluorescence intensities of 750.27 at 1 h and 828.32 at 4 h, suggesting not only successful entry but also retention and accumulation over time. The control samples, in contrast, exhibit no such fluorescence, which validates that the detected signals are attributable to the nanoparticles.

**Figure 5:**
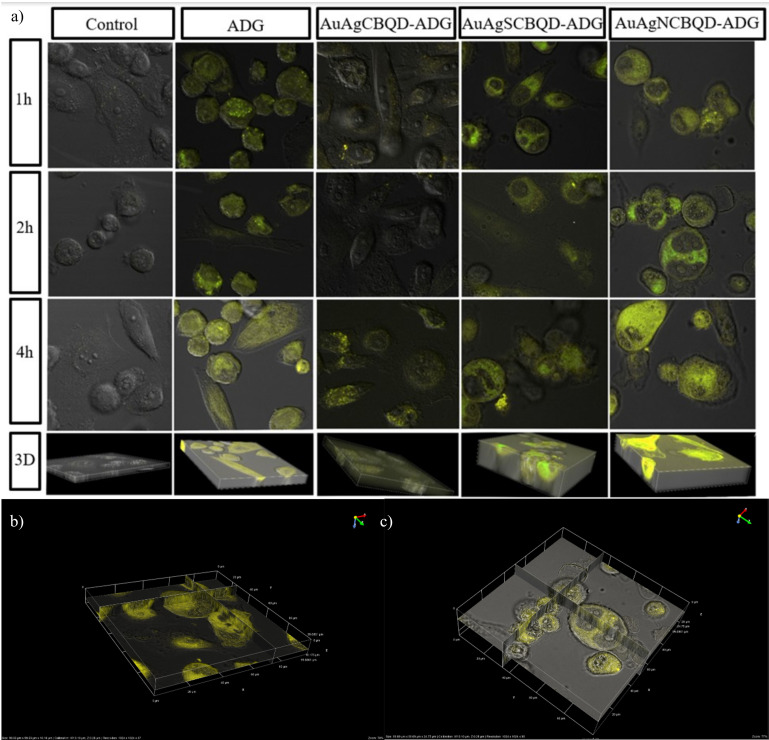
(**a**) Confocal microscopy of PC-3 cells incubated with AuAgCBQD-ADG, AuAgSCBQD-ADG and AuAgNCBQD-ADG at different timeframes. Deconvolution was done via NIS-Elements Offline Deconvolution software. 3D images at 4 h confirm material internalization. 3D virtual sectioning deconvoluted stacked images of PC-3 cells treated with (**b**) AuAgSCBQD and (**c**) AuAgSCBQD-ADG for 2 h. For the rest of the sectioned images see Figure S15.

Figure 5 shows the confocal imaging of the nanoparticles loaded with ADG and the localization patterns seen in Figure 5 further support the hypothesis that the nanoparticles are undergoing cellular uptake mechanisms such as endocytosis. This is particularly evident in localized areas within the cells where there is a concentration of fluorescence, implying that the nanoparticles may be trafficked to specific regions or compartments within the cells. Further evidence of this interpretation are the 3D images, which provide additional spatial context, indicating that the nanoparticles have been internalized rather than simply adhering to the exterior cell surface (see Figures S13 and S14 for non-metal nanoparticle confocal images).

**Figure 6:**
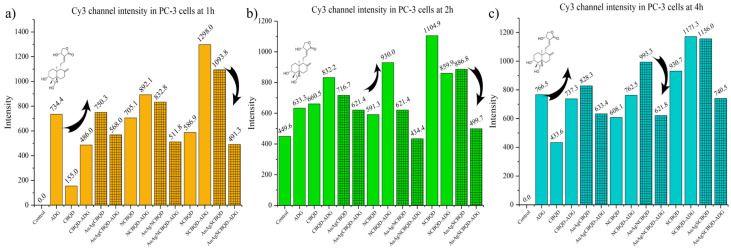
Cy3 channel intensity profiles (with the same threshold) of nanomaterials at: (**a**) 1 h; (**b**) 2 h; (**c**) 4 h. Grid-patterned columns are for the metal nanoparticles.

The confocal microscopy data presents a clear trend of increasing fluorescence intensity over time, which is indicative of the progressive penetration of nanoparticles into the PC-3 cells. Figure 6 shows the intensity of the CBQDs, D-CBQDs, their metal nanocomposites and loaded products grouped by timeframe and compared to the control (PC-3 cells without any material added). It can be observed that in all non-metal nanoparticles, loading them with ADG increased the intensity but in metal nanocomposites, there was a slight quenching observed when loaded with ADG regardless of doping status. This pattern is consistent across various nanoparticles, demonstrating a time-dependent internalization process. For instance, the AuAgNCBQD nanoparticles show a progressive increase in fluorescence intensity, starting from 763.73 at 1 h and reaching up to 993.26 at 4 h. Notably, in most cases, the fluorescence intensity appears to peak at the 4 h time point, which may imply that there is an optimal window for nanoparticle uptake or that saturation levels are being reached within the cells. The absence of such a signal in the control cells serves as an essential baseline, reinforcing that the detected fluorescence is due to the internalization of the nanoparticles and not from any autofluorescence or background noise. This progressive increase in fluorescence intensity is critical for both the basic understanding of nanoparticle–cell interactions and their practical applications. In therapeutic and diagnostic contexts, understanding the kinetics of nanoparticle uptake can influence the timing of drug delivery or the scheduling of imaging procedures.

The 3D stacks and virtual slicing derived from the confocal images offer a more detailed view of how the nanoparticles are distributed within the cellular environment as compared to the 2D images. The patterns observed suggest a primarily cytoplasmic distribution of the nanoparticles. Notably, certain nanoparticles, such as AuAgSCBQD, display a uniform distribution within the cytoplasm with a tendency for perinuclear localization after 2 h of incubation.

This perinuclear accumulation could imply that the nanoparticles are engaging with or are in the vicinity of the cell’s nuclear membrane, which might be indicative of processes like nuclear translocation or interactions with organelles situated near the nucleus, such as the endoplasmic reticulum or the Golgi apparatus. It was observed that the nanocomposite AuAgCBQD directed ADG to the nucleolus as opposed to the CBQD-ADG and the drug alone that dispersed throughout the whole cell. This effect was also observed in the AuAgNCBQDs. In contrast, the SCBQDs and AuAgSCBQDs both directed the drug to more specific areas within the cell than the drug alone. The impact of ADG loading on nanoparticle uptake and detection within PC-3 cells is multifaceted and dependent on a combination of factors, including the physicochemical properties of the nanoparticles and the biological pathways governing cellular internalization and retention. The observed trends underline the importance of considering both the nanoparticle design and the intended cellular environment when developing nanoparticle-based applications. The absence of specific cellular markers in these images means that while the nanoparticles appear to be within the cytoplasm, their presence in specific organelles or the nucleus itself cannot be definitively determined. For instance, without co-staining for organelle-specific proteins, it is not possible to conclusively state whether nanoparticles are within the mitochondria, lysosomes, or other subcellular structures. Thus, these experiments provide a general idea of the nanoparticle–drug localization and behavior for the measured timeframes. In addition, we observe the effect that having a metal core has on drug delivery in comparison to the lone CBQDs and doped-CBQDs.

### 3.6. Surface-Enhanced Raman Spectroscopy

**Figure 7:**
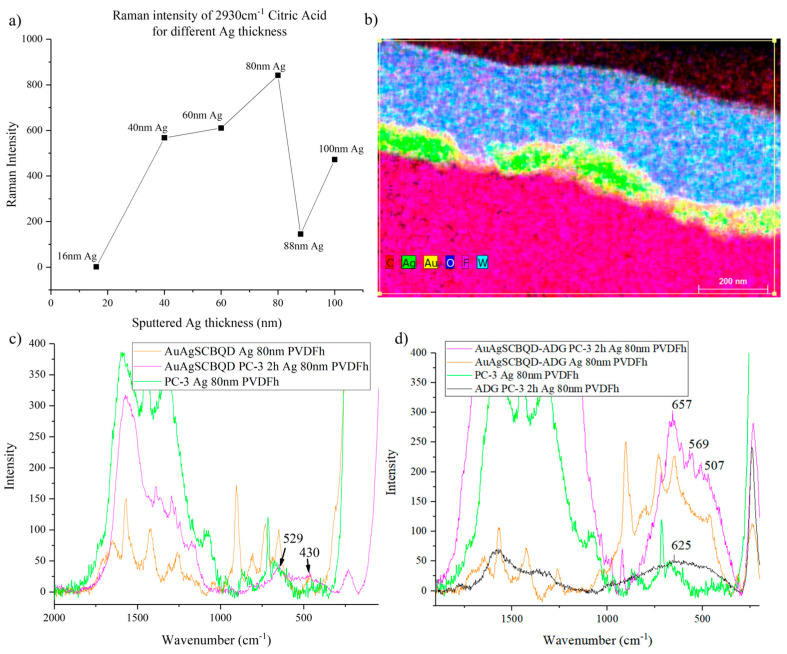
(**a**) Ag thickness vs. intensity of 2930 cm^−1^ peak with 1 µM aqueous solution of citric acid. (**b**) EDS mapping of transversal view of hydrophilic PVDF membrane sputtered with silver. SERS spectra of the PC-3 cells incubated for 2 h with (**c**) AuAgSCBQDs and (**d**) AuAgSCBQD-ADG.

Different thicknesses of silver were evaluated to see which provided the best signal. This was done by adding a drop of an aqueous 1 µM citric acid solution, measuring the 2930 cm^−1^ citric acid Raman peak and comparing the intensities with the thickness of the sputtered silver. Figure 7a shows that 80 nm was the best thickness for signal enhancement using this method. The EDS mapping (Figure 7b) shows the silver present on top of the PVDF membrane along with gold and tungsten from the sample preparation process.

When using the 532 nm excitation source for the Raman measurements on the cells, the laser power needed to be adjusted since 10 mW would burn both the cell and the Ag-sputtered membrane. A laser power of 3 mW was found to be enough to measure the spectra without causing burns. As observed in Figure 7c,d and Figure S17, the SERS spectra of the PC-3 cells treated with the nanomaterials showed distinct signals that were not in the control PC-3 cells’ spectrum. These signals had similarities to the control of the lone nanomaterial’s SERS spectra but often showed shifts. In all cases, when comparing the nanomaterial inside the PC-3 cells with the Andrographolide-loaded nanomaterial, noticeable differences were observed in Raman peak signals. Table S2 summarizes the SERS peak assignments of all the nanomaterials and ADG-loaded products [42]. The most common observation between drug-loaded and non-loaded materials was the presence of the -CH-OH, C-O deformation vibration and ring deformation. Neither of these peaks were present without the loading of the drug. Overall, both unloaded and loaded nanomaterials were observable in a distinguishable manner, indicating that the detection of nanomaterial internalization was successful.

### 3.7. Cell Viability MTS Assays

**Figure 8:**
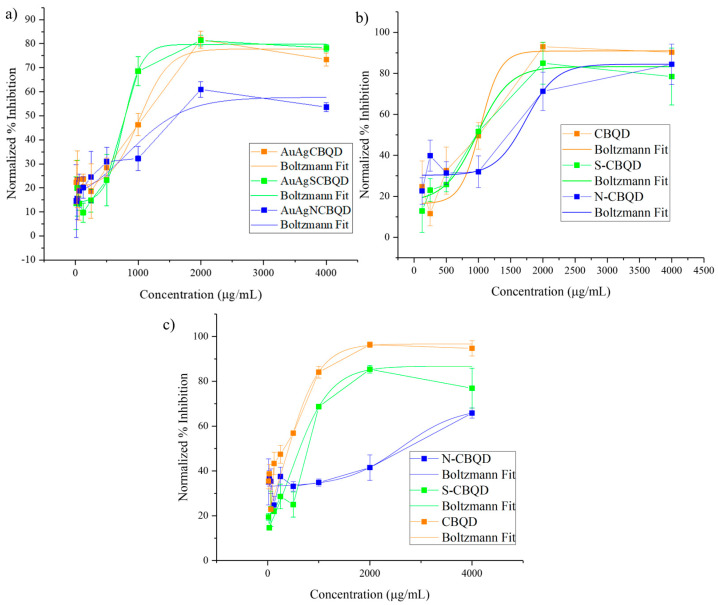
PC-3 cell viability determination using MTS assay for (**a**) AuAgCBQDs, AuAgNCBQDs and AuAgSCBQDs at 48 h of incubation and (**b**) CBQDs, S-CBQDs and N-CBQDs at 48 h of incubation. IC_50_s obtained by solving for x when y equals 50 (percentage of inhibition) in the non-linear fitting of the Boltzmann equation are 1082 μg/mL for AuAgCBQDs, 1574.36 μg/mL for the AuAgNCBQDs and 796.15 μg/mL for the AuAgSCBQDs. CBQDs had a calculated IC_50_ of 999.32 µg/mL, S-CBQDs of 974.76 µg/mL and N-CBQDs of 1604.43 µg/mL. Data sets were validated using ANOVA (results available in Tables S3–S8). (**c**) MTS assay results for CBQDs, S-CBQDs and N-CBQDs after 48 h of incubation in RWPE-1 cells. Data sets were validated using ANOVA (results available in Tables S9–S11). All tests were done in triplicate (n = 3) and *p* values are in PC-3 cells, 0.93 for AuAgCBQDs, 0.554518 for AuAgNCBQDs, 0.80 for AuAgSCBQDs, 0.65 for CBQDs, 0.40 for N-CBQDs, 0.98 for S-CBQDs; for RWPE-1 cells, 0.99 for CBQDs, 0.95 for N-CBQDs and 0.96 for S-CBQDs, showing no statistically significant differences within the sample groups.

The ranges of concentration toxicity above 1000 μg/mL are known to be non-toxic and the range of 500–1000 μg/mL is considered low toxicity [43]. Thus, as seen in Figure 8, the AuAgCBQDs and AuAgNCBQDs are considered non-toxic to PC-3 cells and slightly toxic to AuAgSCBQDs. A literature report shows the IC_50_ of Brazilian red propolis extract gold nanoparticles of 8 nm in size in PC-3 cells to be 53 µg/mL at 24 h [44]. Another study showed that Chinese herbal *Cornus officinalis* extract Ag nanoparticles of 11.7 nm in size had an IC_50_ of 25.54 µg/mL at 48 h [45]. When compared to the IC_50_ results of our nanoparticles and observing the decrease in cytotoxicity, we can conclude with this range of concentrations, including supra-physiological levels, that the CBQD and D-CBQD layers effectively prevent the oxidation of the metal core. The IC_50_ of CBQDs was 406.86 µg/mL, considered moderately toxic. The S-CBQDs had their IC_50_ at 625.20 µg/mL in the range of low toxicity and N-CBQDs had their IC_50_ at 2580.996 µg/mL, showing them to be non-toxic to RWPE-1 cells [43].

## 4. Conclusions

AuAg covered with carbon-based quantum dot nanocomposites were able to reduce the hydrophobicity of ADG down to a Log P of 0.56 without encapsulating the drug. Confocal microscopy suggests primarily cytoplasmic distribution of nanoparticles. The AuAg nanocomposites exhibit a tendency to localize their intracellular distribution more so than the lone CBQDs, N-CBQDs and S-CBQDs. Peaks observed in the SERS spectra confirm that the nanoparticles and ADG enter together into the PC-3 cells, confirming the identity of the fluorophores observed in confocal microscopy in a qualitative and comparative manner. The current SERS data does not explicitly distinguish between nanoparticle-bound ADG and the drug that has been released intracellularly. This is an inherent limitation of SERS-based detection, which reports on molecular proximity rather than the binding state. Regarding the activity and protection against metal core oxidation of the CBQDs, N-CBQDs and S-CBQDs, MTS assays showed that AuAgCBQDs and AuAgNCBQDs are considered non-toxic to PC-3 cells and slightly toxic to AuAgSCBQDs, and that CBQDs and S-CBQDs exhibit low toxicity to PC-3 cells and N-CBQDs are non-toxic to PC-3 cells after 48 h. This shows their stability within a cancerous biosystem. MTS assays on RWPE-1 cells showed that the synthesized CBQDs, N-CBQDs and S-CBQS are biocompatible and good candidates for drug delivery agents. The most biocompatible for RWPE-1 cells is the N-CBQD.

## 5. Patents

Patent (PCT) publication number WO2024206177.

## Figures and Tables

**Table 1:** Measured metal ratio percentages obtained from EDS of gold–silver alloy nanocomposites. See Supporting Information for EDS spectrum and element quantification (Figures S1–S3).

Name	Au wt. %	Error (±) in wt. %	Au Ratio (%)	Ag wt. %	Error (±) in wt. %	Ag Ratio (%)
AuAgCBQD	15.61	5.01	37.51	26.02	8.03	62.49
AuAgNCBQD	11.18	3.60	40.74	16.26	5.05	59.26
AuAgSCBQD	5.85	1.96	31.94	19.58	6.03	68.06

## Data Availability

The original contributions presented in this study are included in the article/Supplementary Material. Further inquiries can be directed at the corresponding author(s).

## Supplementary Materials

The following supporting information can be downloaded at: https://www.mdpi.com/article/10.3390/nano16070396/s1. Figure S1: EDS spectrum and element quantification of AuAgCBQD; Figure S2: EDS spectrum and element quantification of AuAgNCBQD; Figure S3: EDS spectrum and element quantification of AuAgSCBQD; Figure S4: ^1^H-NMR spectra of Andrographolide (ADG) in D_2_O; Figure S5: ^1^H-NMR spectra of AuAgCBQD in D_2_O; Figure S6: ^1^H-NMR spectra of AuAgCBQD-ADG in D_2_O. Figure S7: ^1^H-NMR spectra of AuAgNCBQD in D_2_O; Figure S8: ^1^H-NMR spectra of AuAgNCBQD-ADG in D_2_O; Figure S9: ^1^H-NMR spectra of AuAgSCBQD in D_2_O; Figure S10: ^1^H-NMR spectra of AuAgSCBQD-ADG in D_2_O; Figure S11: (Left) Emission spectra of AuAgCBQDs, AuAgNCBQDs and AuAgSCBQDs. (Right) Emission spectra of CBQDs, N-CBQDs and S-CBQDs; Figure S12: Excitation and emission spectra at different known physiological pHs present in cancer of (a) CBQDs, (b) CBQD-ADG, (c) N-CBQD, (d) NCBQD-ADG, (e) S-CBQD, (f) SCBQD-ADG; Figure S13: Confocal microscopy of PC-3 cells incubated with CBQD, S-CBQD and N-CBQD at different timeframes; Figure S14: Confocal microscopy of PC-3 cells incubated with CBQD-ADG, SCBQD-ADG and NCBQD-ADG at different timeframes; Table S1: Confocal Microscopy Observations; Figure S15: 3D virtual sectioning deconvoluted stacked images of PC-3 cells treated with nanomaterials for 2 h. (a) Control, (b) ADG, (c) CBQD, (d) CBQD-ADG, (e) N-CBQD, (f) NCBQD-ADG, (g) S-CBQD, (h) SCBQD-ADG, (i) AuAgCBQD, (j) AuAgCBQD-ADG, (k) AuAgNCBQD and l) AuAgNCBQD-ADG; Figure S16: In situ EDS spectra of Ag sputtered hydrophilic PVDF membrane; Figure S17: SERS spectra of the PC-3 cells incubated for 2 h with (a) CBQDs, (b) CBQD-ADG, (c) N-CBQDs, (d) NCBQD-ADG, (e) AuAgCBQDs, (f) AuAgCBQD-ADG, (g) AuAgNCBQD, (h) AuAgNCBQD-ADG; Table S2: Summary of SERS peaks within the PC-3 cells after 2 h incubation; Table S3: ANOVA single factor for AuAgCBQDs in PC-3 cells; Table S4: ANOVA single factor for AuAgNCBQDs in PC-3 cells; Table S5: ANOVA single factor for AuAgSCBQDs in PC-3 cells; Table S6: ANOVA single factor for CBQDs in PC-3 cells; Table S7: ANOVA single factor for N-CBQDs in PC-3 cells; Table S8: ANOVA single factor for S-CBQDs in PC-3 cells; Table S9: ANOVA single factor for CBQDs in RWPE-1 cells; Table S10: ANOVA single factor for N-CBQDs in RWPE-1 cells; Table S11: ANOVA single factor for S-CBQDs in RWPE-1 cells.

## Abbreviations

The following abbreviations are used in this manuscript:

Log P	Partition coefficient
PC-3	Cell line initiated from a bone metastasis of a grade IV prostatic adenocarcinoma from a 62-year-old, White, male.
RWPE-1	An epithelial cell that was isolated from the prostate of a White, 54-year-old, male patient.
SERS	Surface-enhanced Raman spectroscopy
MTS assay	3-(4,5-dimethylthiazol-2-yl)-5-(3-carboxymethoxyphenyl)-2-(4-sulfophenyl)-2H-tetrazolium assay
CBQDs	Carbon-based quantum dots
D-CBQDs	Doped carbon-based quantum dots
N-CBQDs	Nitrogen-doped carbon-based quantum dots
S-CBQDs	Sulfur-doped carbon-based quantum dots
ADG	Andrographolide
CME	Caveolae-mediated endocytosis
AuAgCBQD	Gold–silver alloy nanoparticle covered with carbon-based quantum dots
AuAgNCBQD	Gold–silver alloy nanoparticle covered with nitrogen doped carbon-based quantum dots
AuAgSCBQD	Gold–silver alloy nanoparticle covered with sulfur-doped carbon-based quantum dots
NMR	Nuclear Magnetic Resonance
TEM	Transmission Electron Microscopy
EDS	Energy dispersive X-ray analysis
SEM	Scanning electron microscopy
UV-Vis	Ultraviolet–visible
RI_W_^init^	Relative NMR signal integration of the materials in D_2_O before equilibration with 1-octanol
RI_W_^equil^	Relative NMR signal integration of the materials in the aqueous phase after equilibration
K_ow_	1-octanol/water equilibrium constant
FBS	Fetal bovine serum
PBS	Phosphate-buffered saline
PVDFh	Hydrophilic polyvinylidene fluoride
ANOVA	Analysis of variance
wt.%	Weight percentage
ppm	Parts per million
NOE	Nuclear Overhauser Effect
IC_50_	Half-maximal inhibitory concentration

## References

[1] Habiba K., Encarnacion-Rosado J., Garcia-Pabon K., Villalobos-Santos J.C., Makarov V.I., Avalos J.A., Weiner B.R., Morell G. (2015). Improving Cytotoxicity against Cancer Cells by Chemo-Photodynamic Combined Modalities Using Silver-Graphene Quantum Dots Nanocomposites. Int. J. Nanomed..

[2] Zhang L., Liu L., Wang J., Niu M., Zhang C., Yu S., Yang Y. (2020). Functionalized Silver Nanoparticles with Graphene Quantum Dots Shell Layer for Effective Antibacterial Action. J. Nanopart Res..

[3] Farokhnezhad M., Esmaeilzadeh M. (2019). Optical and Photothermal Properties of Graphene Coated Au–Ag Hollow Nanoshells: A Modeling for Efficient Photothermal Therapy. J. Phys. Chem. C.

[4] Liu J., Zhai F., Zhou H., Yang W., Zhang S. (2019). Nanogold Flower-Inspired Nanoarchitectonics Enables Enhanced Light-to-Heat Conversion Ability for Rapid and Targeted Chemo-Photothermal Therapy of a Tumor. Adv. Healthcare Mater..

[5] Chen W., Shen J., Chen S., Yan J., Zhang N., Zheng K., Liu X. (2019). Synthesis of Graphene Quantum Dot-Stabilized Gold Nanoparticles and Their Application. RSC Adv..

[6] Jacob S., Rao R., Gorain B., Boddu S.H.S., Nair A.B. (2025). Solid Lipid Nanoparticles and Nanostructured Lipid Carriers for Anticancer Phytochemical Delivery: Advances, Challenges, and Future Prospects. Pharmaceutics.

[7] Chugh H., Sood D., Chandra I., Tomar V., Dhawan G., Chandra R. (2018). Role of Gold and Silver Nanoparticles in Cancer Nano-Medicine. Artif. Cells Nanomed. Biotechnol..

[8] Hogarth C., Arnold K., McLauchlin A., Rannard S.P., Siccardi M., McDonald T.O. (2021). Evaluating the Impact of Systematic Hydrophobic Modification of Model Drugs on the Control, Stability and Loading of Lipid-Based Nanoparticles. J. Mater. Chem. B.

[9] Forestier-Román I.S., López-Rivas A., Sánchez-Vázquez M.M., Rohena-Rivera K., Nieves-Burgos G., Ortiz-Zuazaga H., Torres-Ramos C.A., Martínez-Ferrer M. (2019). Andrographolide Induces DNA Damage in Prostate Cancer Cells. Oncotarget.

[10] Pandey G., Rao C. (2018). Andrographolide: Its Pharmacology, Natural Bioavailability and Current Approaches to Increase Its Content in Andrographispaniculata. Int. J. Complement. Altern. Med..

[11] Schulte B., König M., Escher B.I., Wittenburg S., Proj M., Wolf V., Lemke C., Schnakenburg G., Sosič I., Streeck H. (2022). Andrographolide Derivatives Target the KEAP1/NRF2 Axis and Possess Potent Anti-SARS-CoV-2 Activity. ChemMedChem.

[12] Chandrasekaran B., Abed S.N., Al-Attraqchi O., Kuche K., Tekade R.K. (2018). Computer-Aided Prediction of Pharmacokinetic (ADMET) Properties. Dosage Form Design Parameters.

[13] Augustine R., Hasan A., Primavera R., Wilson R.J., Thakor A.S., Kevadiya B.D. (2020). Cellular Uptake and Retention of Nanoparticles: Insights on Particle Properties and Interaction with Cellular Components. Mater. Today Commun..

[14] Pillay V., Murugan K., Choonara Y.E., Kumar P., Bijukumar D., Du Toit L.C. (2015). Parameters and Characteristics Governing Cellular Internalization and Trans-Barrier Trafficking of Nanostructures. Int. J. Nanomed..

[15] Hill M.M., Bastiani M., Luetterforst R., Kirkham M., Kirkham A., Nixon S.J., Walser P., Abankwa D., Oorschot V.M.J., Martin S. (2008). PTRF-Cavin, a Conserved Cytoplasmic Protein Required for Caveola Formation and Function. Cell.

[16] Watson P. (2005). Intracellular Trafficking Pathways and Drug Delivery: Fluorescence Imaging of Living and Fixed Cells. Adv. Drug Deliv. Rev..

[17] Man F., Gawne P.J., De Rosales R.T.M. (2019). Nuclear Imaging of Liposomal Drug Delivery Systems: A Critical Review of Radiolabelling Methods and Applications in Nanomedicine. Adv. Drug Deliv. Rev..

[18] Mandal S., Zhou Y., Shibata A., Destache C.J. (2015). Confocal Fluorescence Microscopy: An Ultra-Sensitive Tool Used to Evaluate Intracellular Antiretroviral Nano-Drug Delivery in HeLa Cells. AIP Adv..

[19] Vanden-Hehir S., Tipping W., Lee M., Brunton V., Williams A., Hulme A. (2019). Raman Imaging of Nanocarriers for Drug Delivery. Nanomaterials.

[20] Agarwal A., Tripathi P., Tripathi S., Jain N. (2008). Fluorescence Imaging: Applications in Drug Delivery Research. Curr. Drug Targets.

[21] Littleford R.E., Graham D., Smith W.E., Khan I. (2017). Surface-Enhanced Raman Scattering (SERS), Applications. Encyclopedia of Spectroscopy and Spectrometry.

[22] Yilmaz H., Yilmaz D., Taskin I.C., Culha M. (2022). Pharmaceutical Applications of a Nanospectroscopic Technique: Surface-Enhanced Raman Spectroscopy. Adv. Drug Deliv. Rev..

[23] Schneider C.A., Rasband W.S., Eliceiri K.W. (2012). NIH Image to ImageJ: 25 Years of Image Analysis. Nat. Methods.

[24] Medina-Berríos N., Pantoja-Romero W., Lavin Flores A., Díaz-Vélez S.C., Martínez-Guadalupe A.C., Torres-Mulero M.T., Kisslinger K., Martínez-Ferrer M., Morell G., Weiner B.R. (2024). Synthesis and Characterization of Carbon-Based Quantum Dots and Doped Derivatives for Improved Andrographolide’s Hydrophilicity in Drug Delivery Platforms. ACS Omega.

[25] Nozaki T., Kakuda T., Pottathara Y.B., Kawasaki H. (2019). A Nanocomposite of N-Doped Carbon Dots with Gold Nanoparticles for Visible Light Active Photosensitisers. Photochem. Photobiol. Sci..

[26] Huhtamäki T., Tian X., Korhonen J.T., Ras R.H.A. (2018). Surface-Wetting Characterization Using Contact-Angle Measurements. Nat. Protoc..

[27] Krainer S., Hirn U. (2021). Contact Angle Measurement on Porous Substrates: Effect of Liquid Absorption and Drop Size. Colloids Surf. A Physicochem. Eng. Asp..

[28] Gallardo-Moreno A.M., Navarro-Pérez M.L., Vadillo-Rodríguez V., Bruque J.M., González-Martín M.L. (2011). Insights into Bacterial Contact Angles: Difficulties in Defining Hydrophobicity and Surface Gibbs Energy. Colloids Surf. B Biointerfaces.

[29] Pearce A.K., O’Reilly R.K. (2021). Polymers for Biomedical Applications: The Importance of Hydrophobicity in Directing Biological Interactions and Application Efficacy. Biomacromolecules.

[30] Cumming H., Rücker C. (2017). Octanol–Water Partition Coefficient Measurement by a Simple ^1^ H NMR Method. ACS Omega.

[31] Zwolak J.W., Boggs P.T., Watson L.T. (2007). Algorithm 869: ODRPACK95: A Weighted Orthogonal Distance Regression Code with Bound Constraints. ACM Trans. Math. Softw..

[32] Attota R.K., Liu E.C. (2016). Volume Determination of Irregularly-Shaped Quasi-Spherical Nanoparticles. Anal. Bioanal. Chem..

[33] Aygun A., Cobas I., Tiri R.N.E., Sen F. (2024). Hydrothermal Synthesis of B, S, and N-Doped Carbon Quantum Dots for Colorimetric Sensing of Heavy Metal Ions. RSC Adv..

[34] Kline T.P., Marzilli L.G., Live D., Zon G. (1990). NMR Studies of an Oligonucleotide with an Unusual Structure Induced by Platinum Anticancer Drugs. Biochem. Pharmacol..

[35] Wang Y., Li M., Xu L., Tang T., Ali Z., Huang X., Hou Y., Zhang S. (2019). Polar and Conductive Iron carbide@N-Doped Porous Carbon Nanosheets as a Sulfur Host for High Performance Lithium Sulfur Batteries. Chem. Eng. J..

[36] Kou X., Jiang S., Park S.-J., Meng L.-Y. (2020). A Review: Recent Advances in Preparations and Applications of Heteroatom-Doped Carbon Quantum Dots. Dalton Trans..

[37] Huggins D.J., Sherman W., Tidor B. (2012). Rational Approaches to Improving Selectivity in Drug Design. J. Med. Chem..

[38] Bilardo R., Traldi F., Vdovchenko A., Resmini M. (2022). Influence of Surface Chemistry and Morphology of Nanoparticles on Protein Corona Formation. WIREs Nanomed. Nanobiotechnol..

[39] Wang S., Nishiuchi T., Pignedoli C.A., Yao X., Di Giovannantonio M., Zhao Y., Narita A., Feng X., Müllen K., Ruffieux P. (2022). Steering On-Surface Reactions through Molecular Steric Hindrance and Molecule-Substrate van Der Waals Interactions. Quantum Front..

[40] Anderson M., Moshnikova A., Engelman D.M., Reshetnyak Y.K., Andreev O.A. (2016). Probe for the Measurement of Cell Surface pH in Vivo and Ex Vivo. Proc. Natl. Acad. Sci. USA.

[41] Li Z., He P., Luo G., Shi X., Yuan G., Zhang B., Seidl C., Gewies A., Wang Y., Zou Y. (2020). Increased Tumoral Microenvironmental pH Improves Cytotoxic Effect of Pharmacologic Ascorbic Acid in Castration-Resistant Prostate Cancer Cells. Front. Pharmacol..

[42] Socrates G. (2010). Infrared and Raman Characteristic Group Frequencies: Tables and Charts.

[43] De Lima R.M.T., Dos Reis A.C., De Oliveira Santos J.V., De Oliveira Ferreira J.R., Lima Braga A., De Oliveira Filho J.W.G., De Menezes A.-A.P.M., Da Mata A.M.O.F., De Alencar M.V.O.B., Do Nascimento Rodrigues D.C. (2019). Toxic, Cytogenetic and Antitumor Evaluations of [6]-Gingerol in Non-Clinical in Vitro Studies. Biomed. Pharmacother..

[44] Botteon C.E.A., Silva L.B., Ccana-Ccapatinta G.V., Silva T.S., Ambrosio S.R., Veneziani R.C.S., Bastos J.K., Marcato P.D. (2021). Biosynthesis and Characterization of Gold Nanoparticles Using Brazilian Red Propolis and Evaluation of Its Antimicrobial and Anticancer Activities. Sci. Rep..

[45] He Y., Li X., Wang J., Yang Q., Yao B., Zhao Y., Zhao A., Sun W., Zhang Q. (2017). Synthesis, Characterization and Evaluation Cytotoxic Activity of Silver Nanoparticles Synthesized by Chinese Herbal Cornus Officinalis via Environment Friendly Approach. Environ. Toxicol. Pharmacol..

