# Supplementary material for: AuAg–Carbon-Based Quantum Dot Nanocomposites to Reduce Andrographolide’s Hydrophobicity and Drug Internalization Tracking in PC-3 Cells
Source: Nanomaterials (Basel). 2026 Mar 25;16(7):396. doi: 10.3390/nano16070396 (PMC13075103; doi:10.3390/nano16070396)
Supplement: Supplementary file 1 [file nanomaterials-16-00396-s001.zip › nanomaterials-4114071-supplementary.pdf]

Supporting information

AuAg-Carbon Based Quantum Dot nanocomposites to reduce Andrographolide's hydrophobicity and drug internalization tracking in PC-3 cells

Nataniel Medina-Berríos[a,b], Alondra Veloz-Bonilla[b,c], Sebastián C. Díaz Vélez[a,b], Mariana T. Torres Mulero[b,c], Kim Kisslinger[d], Alejandro O. Rivera-Torres[c,e], Gerardo Morell[b,f], Magaly Martínez-Ferrer[e,g] & Brad R. Weiner[a,b]*

[a]Department of Chemistry, University of Puerto Rico, Rio Piedras Campus, San Juan, PR.

[b]Molecular Sciences Research Center, University of Puerto Rico, San Juan, PR.

[c]Department of Biology, University of Puerto Rico, Rio Piedras Campus, San Juan, PR.

[d]Brookhaven National Lab, Upton, NY

[e] University of Puerto Rico Comprehensive Cancer Center, Division of Cancer Biology, San Juan, PR.

[f]Department of Physics, University of Puerto Rico, Rio Piedras Campus, San Juan, PR.

[g]Department of Pharmaceutical Sciences, School of Pharmacy, Medical Sciences Campus, University of Puerto Rico, San Juan, PR.

* Correspondence: nataniel.medina@upr.edu

Keywords: nanoparticle, nanocomposite, carbon-based quantum dots, gold-silver alloy, drug delivery, hydrophobic drugs, prostate cancer.

Table of contents

Figure S1: EDS spectrum and element quantification of AuAgCBQD.

Figure S2: EDS spectrum and element quantification of AuAgNCBQD

Figure S3: EDS spectrum and element quantification of AuAgSCBQD.

Figure S4: ^{1}H-NMR spectra of Andrographolide (ADG) in D_2O.

Figure S5: ^{1}H-NMR spectra of AuAgCBQD in D_2O.

Figure S6: ^{1}H-NMR spectra of AuAgCBQD-ADG in D_2O.

Figure S7: 1H-NMR spectra of AuAgNCBQD in D_2O.

Figure S8: ^{1}H-NMR spectra of AuAgNCBQD-ADG in D_2O.

Figure S9: ^{1}H-NMR spectra of AuAgSCBQD in D_2O.

Figure S10: ^{1}H-NMR spectra of AuAgSCBQD-ADG in D_2O.

Figure S11: (Left) Emission spectra of AuAgCBQDs, AuAgNCBQDs and AuAgSCBQDs. (Right) Emission spectra of CBQDs, N-CBQDs and S-CBQDs.

Figure S12: Excitation and emission spectra at different known physiological pHs present in cancer of a) CBQDs, b) CBQD-ADG, c) N-CBQD, d) NCBQD-ADG, e) S-CBQD, f) SCBQD-ADG.

Figure S13: Confocal microscopy of PC-3 cells incubated with CBQD, S-CBQD and N-CBQD at different timeframes.

Figure S14: Confocal microscopy of PC-3 cells incubated with CBQD-ADG, SCBQD-ADG and NCBQD-ADG at different timeframes.

Table S1: Confocal Microscopy Observations

Figure S15: 3D virtual sectioning deconvoluted stacked images of PC-3 cells treated with nanomaterials for 2 h. a) Control, b) ADG, c) CBQD, d) CBQD-ADG, e) N-CBQD, f) NCBQD-ADG, g) S-CBQD, h) SCBQD-ADG, i) AuAgCBQD, j) AuAgCBQD-ADG, k) AuAgNCBQD and l) AuAgNCBQD-ADG.

Figure S16: In-situ EDS spectra of Ag sputtered hydrophilic PVDF membrane.

Figure S17: SERS spectra of the PC-3 cells incubated for 2 h with a) CBQDs, b) CBQD-ADG, c) N-CBQDs, d) NCBQD-ADG, e) AuAgCBQDs, f) AuAgCBQD-ADG, g) AuAgNCBQD & h) AuAgNCBQD-ADG.

Table S2: Summary of SERS peaks within the PC-3 cells after 2 h incubation.

Table S3: Anova Single Factor for AuAgCBQDs in PC-3 cells

Table S4: Anova Single Factor for AuAgNCBQDs in PC-3 cells

Table S5: Anova Single Factor for AuAgSCBQDs in PC-3 cells

Table S6: ANOVA Single Factor for CBQDs in PC-3 cells

Table S7: ANOVA Single Factor for N-CBQDs in PC-3 cells

Table S8: ANOVA Single Factor for S-CBQDs in PC-3 cells

Table S9: ANOVA Single Factor for CBQDs in RWPE-1 cells

Table S10: ANOVA Single Factor for N-CBQDs in RWPE-1 cells

Table S11: ANOVA Single Factor for S-CBQDs in RWPE-1 cells

In-situ EDS Spectra

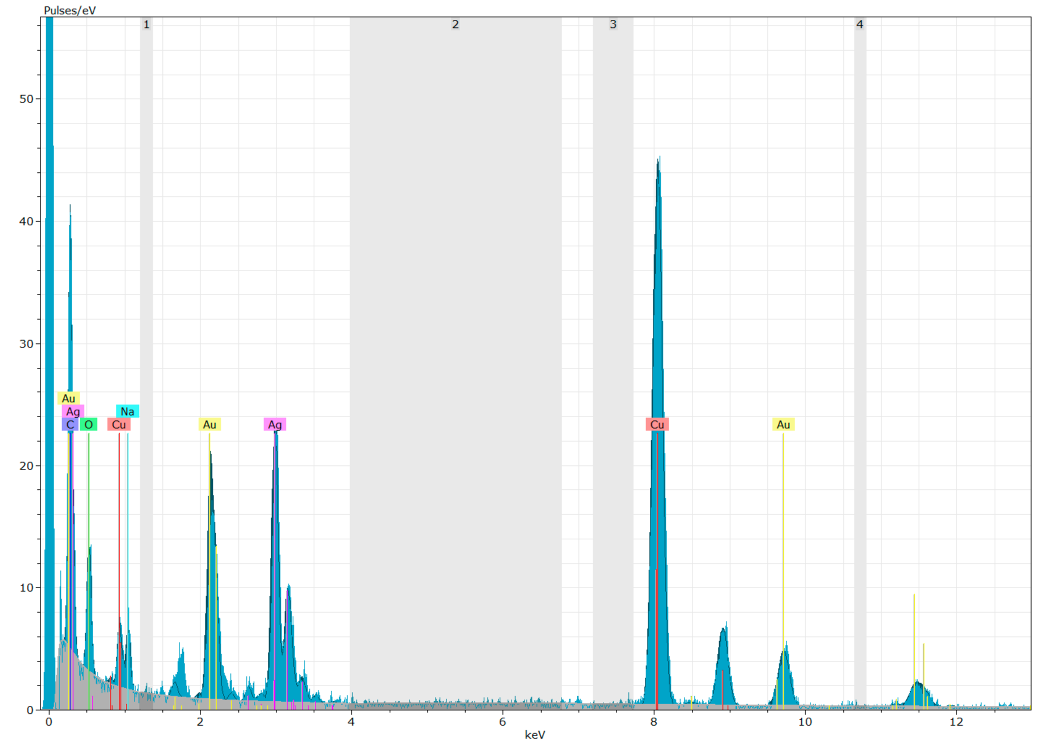

Element	series	Net	[wt.%]	[norm. wt.%]	[norm. at.%]	Error in wt.% (3 Sigma)
Copper (Cu)	K-series	8165	44.60997099	44.60997099	33.72347587	4.391981
Oxygen (O)	K-series	673	2.15088113	2.15088113	6.458048875	0.390183
Carbon (C)	K-series	1937	10.55684023	10.55684023	42.2224469	1.266977
Sodium (Na)	K-series	380	1.053986133	1.053986133	2.202362953	0.262906
Silver (Ag)	L-series	4084	26.01538995	26.01538995	11.58580772	8.02711
Gold (Au)	L-series	1569	15.61293156	15.61293156	3.807857674	5.018137
		Sum:	100	100	100	

Figure S1: EDS spectrum and element quantification of AuAgCBQD.

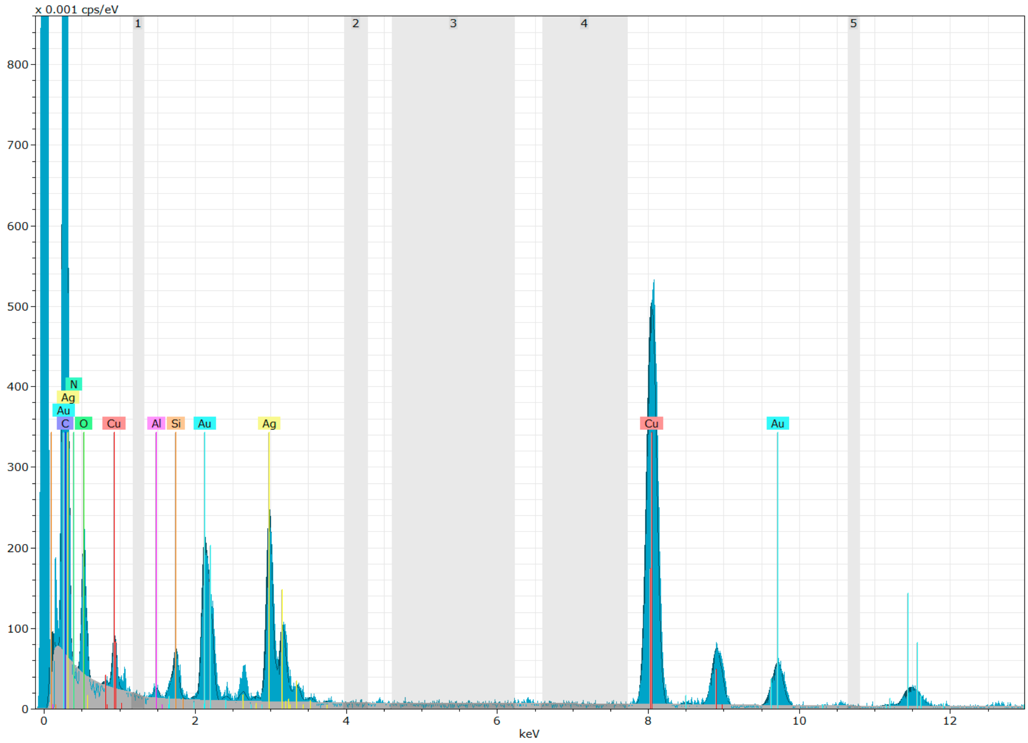

Element	series	Net	[wt.%]	[norm. wt.%]	[norm. at.%]	Error in wt.% (3 Sigma)
Copper (Cu)	K-series	8370	30.19786	30.19786	11.58823	2.992425
Oxygen (O)	K-series	1007	2.12273	2.12273	3.235341	0.352147
Carbon (C)	K-series	10794	38.86392	38.86392	78.90355	3.748354
Gold (Au)	L-series	1698	11.17594	11.17594	1.383632	3.5993
Aluminium (Al)	K-series	102	0.223552	0.223552	0.202042	0.144386
Silver (Ag)	L-series	3864	16.25869	16.25869	3.675546	5.049934
Silicon (Si)	K-series	529	1.14949	1.14949	0.99805	0.228001
Nitrogen (N)	K-series	2	0.00782	0.00782	0.013614	0.088562
		Sum:	100	100	100	

Figure S2: EDS spectrum and element quantification of AuAgNCBQD

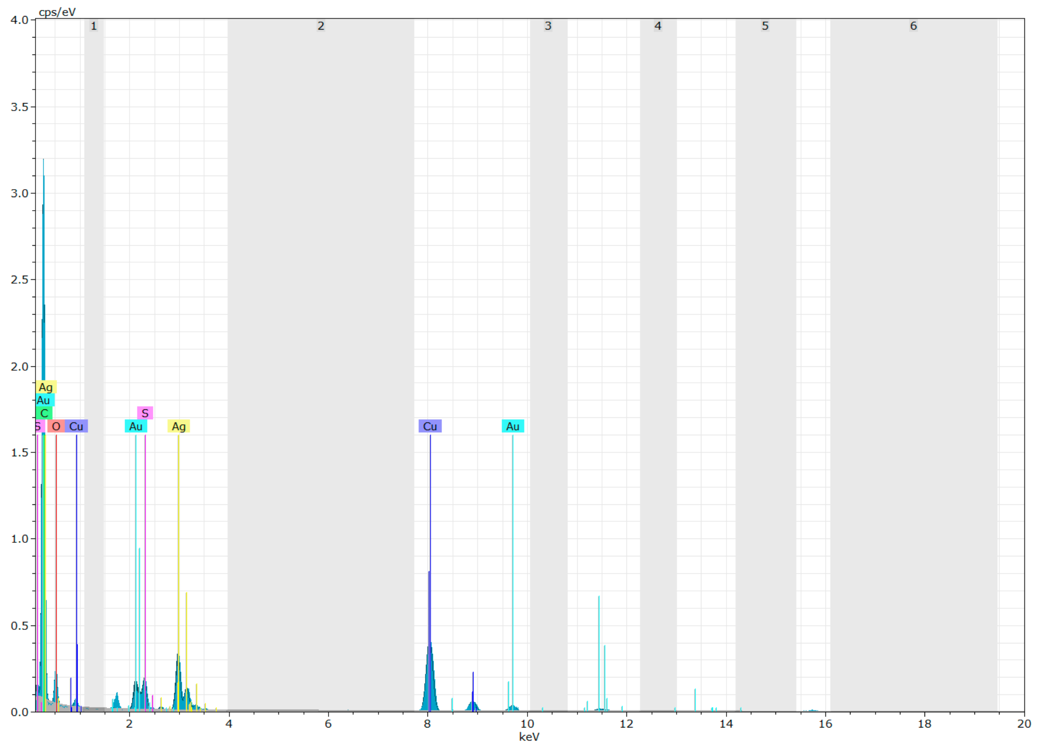

Element	series	Net	[wt.%]	[norm. wt.%]	[norm. at.%]	Error in wt.% (3 Sigma)
Oxygen (O)	K-series	1283	2.114119	2.114119407	2.808715152	0.334915
Carbon (C)	K-series	16703	46.95681	46.95681012	83.10005433	4.439422
Copper (Cu)	K-series	7890	22.23104	22.23103509	7.436239155	2.231355
Gold (Au)	L-series	1139	5.851914	5.851914205	0.631520539	1.962952
Sulfur (S)	K-series	1942	3.266252	3.266251538	2.165143644	0.447861
Silver (Ag)	L-series	5960	19.57987	19.57986964	3.858327181	6.025229
		Sum:	100	100	100	

Figure S3: EDS spectrum and element quantification of AuAgSCBQD.

NMR Spectroscopy

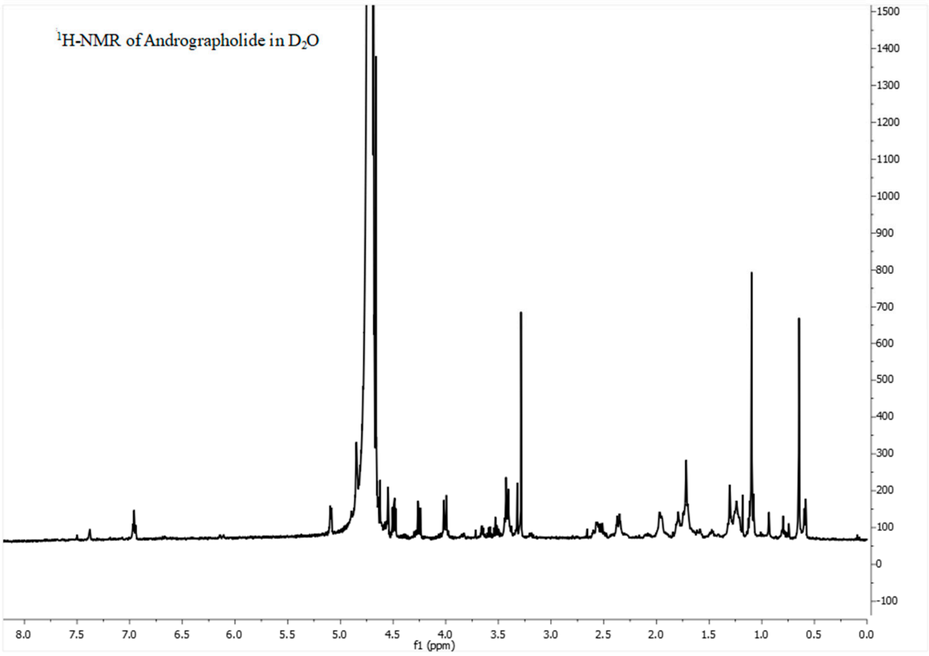

Figure S4: ^{1}H-NMR spectra of Andrographolide (ADG) in D_2O.

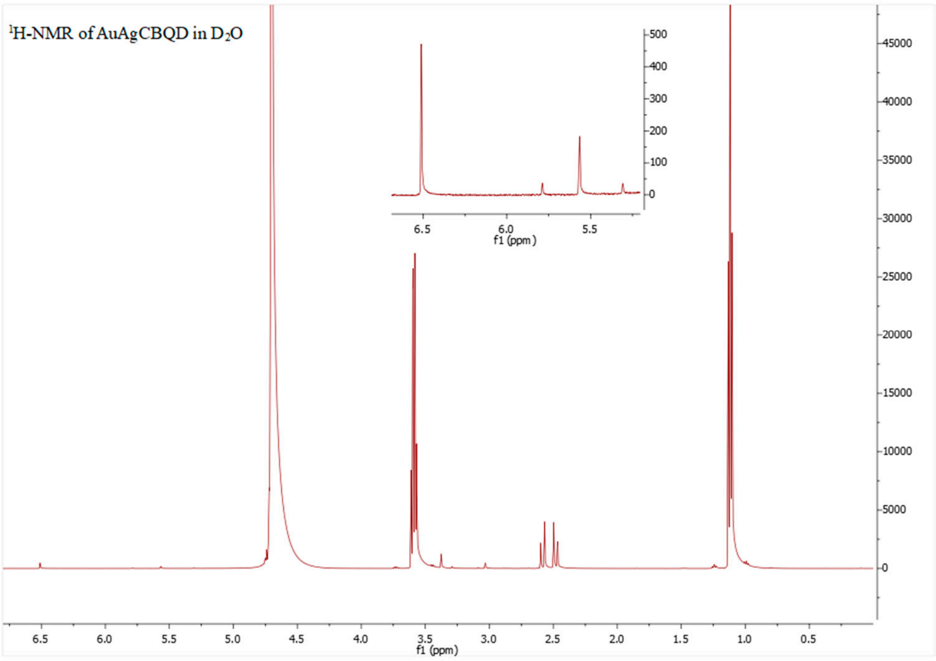

Figure S5: ^{1}H-NMR spectra of AuAgCBQD in D_2O.

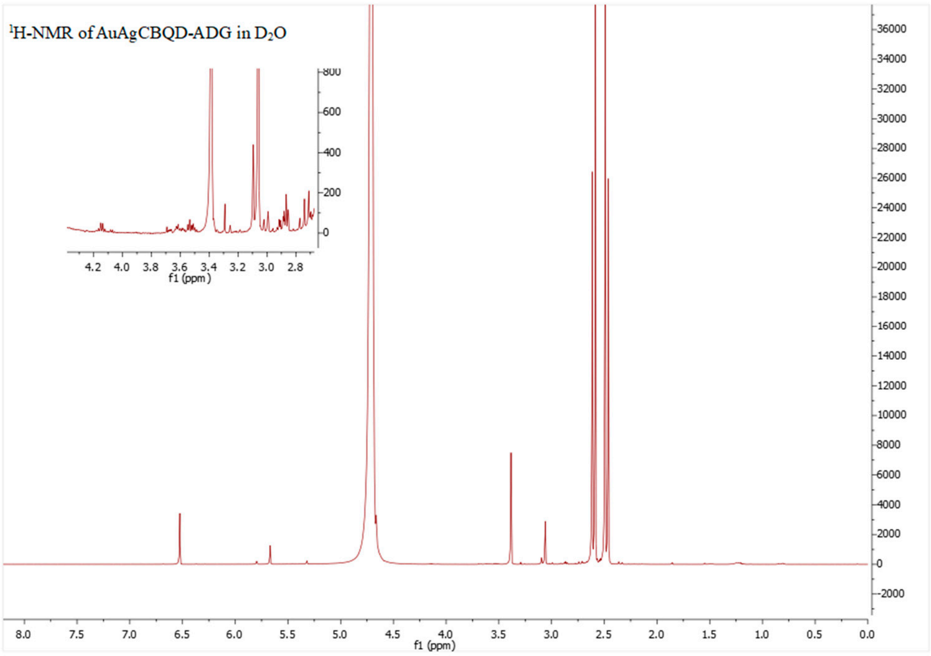

Figure S6: ^{1}H-NMR spectra of AuAgCBQD-ADG in D_2O.

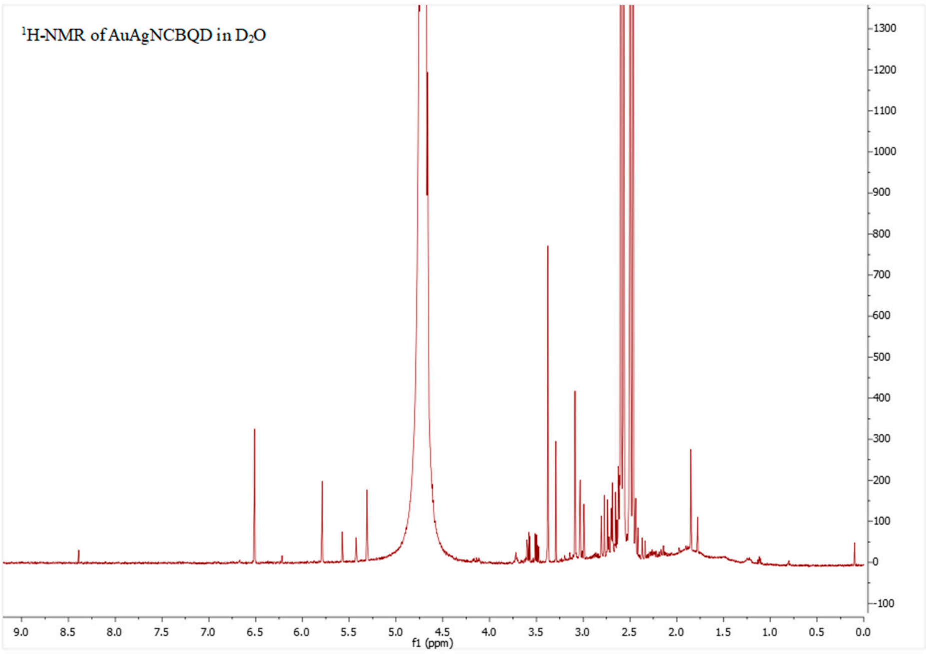

Figure S7: ^{1}H-NMR spectra of AuAgNCBQD in D_2O.

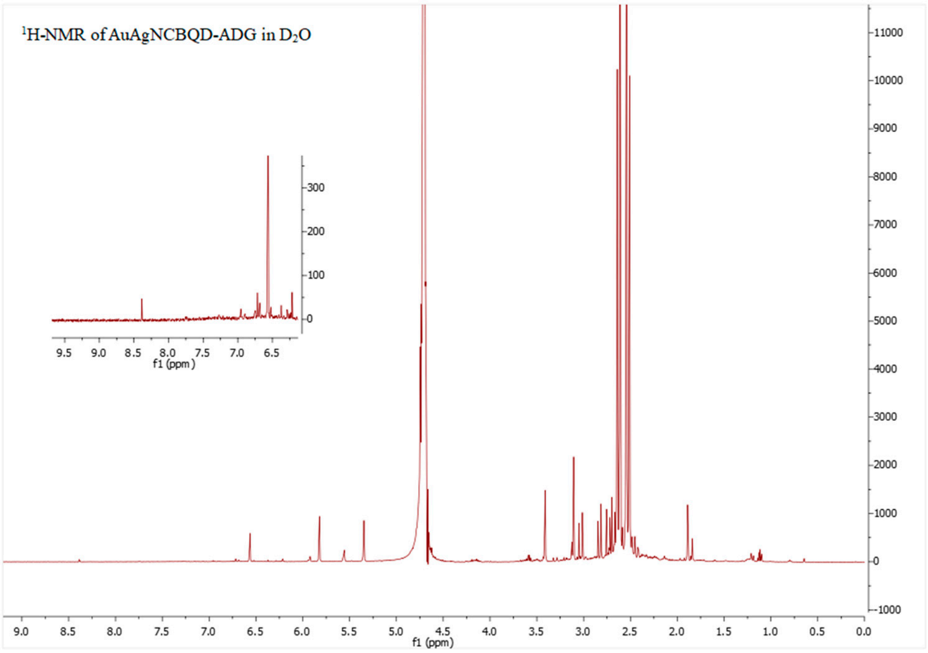

Figure S8: ^{1}H-NMR spectra of AuAgNCBQD-ADG in D_2O.

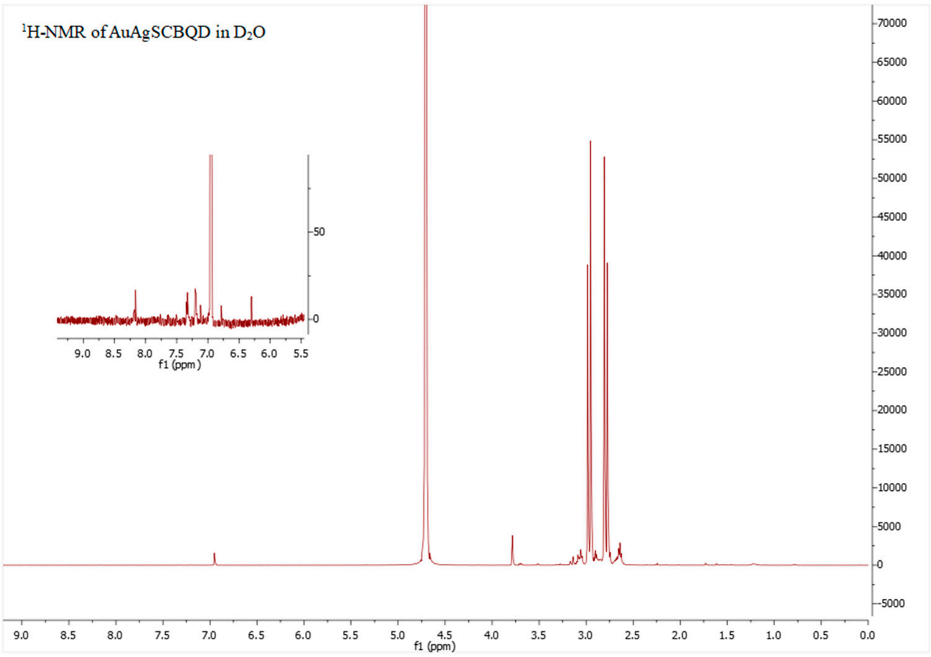

Figure S9: ^{1}H-NMR spectra of AuAgSCBQD in D_2O.

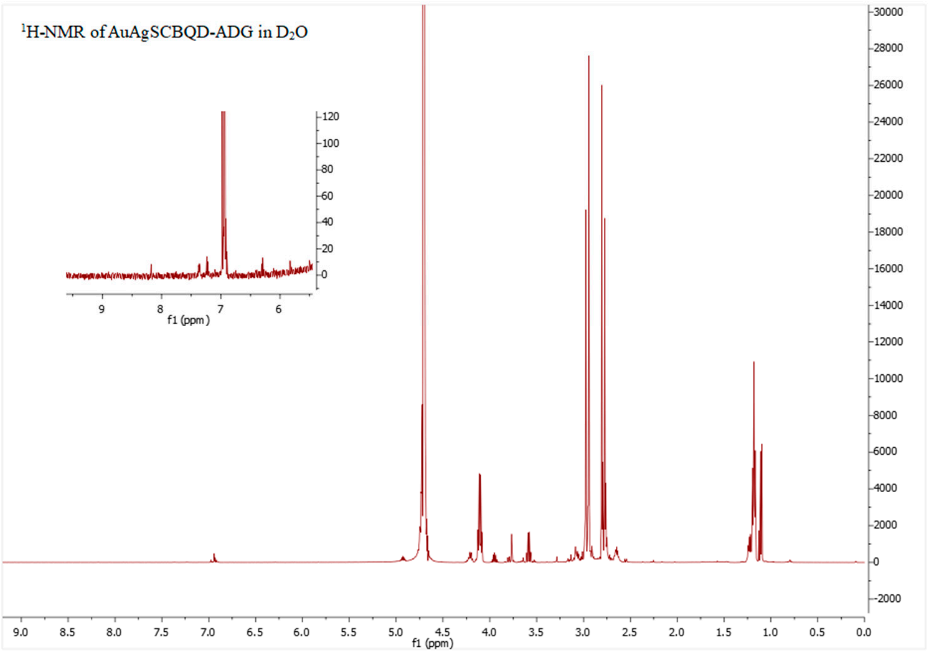

Figure S10: ^{1}H-NMR spectra of AuAgSCBQD-ADG in D_2O.

Fluorescence Spectroscopy

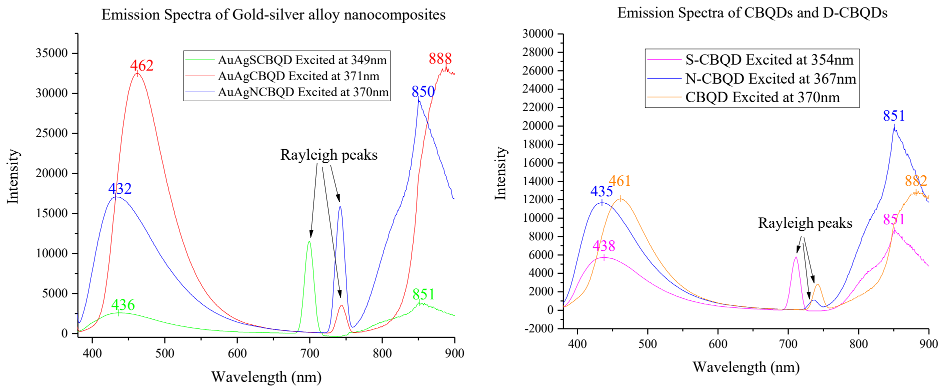

Figure S11: (Left) Emission spectra of AuAgCBQDs, AuAgNCBQDs and AuAgSCBQDs.

(Right) Emission spectra of CBQDs, N-CBQDs and S-CBQDs.

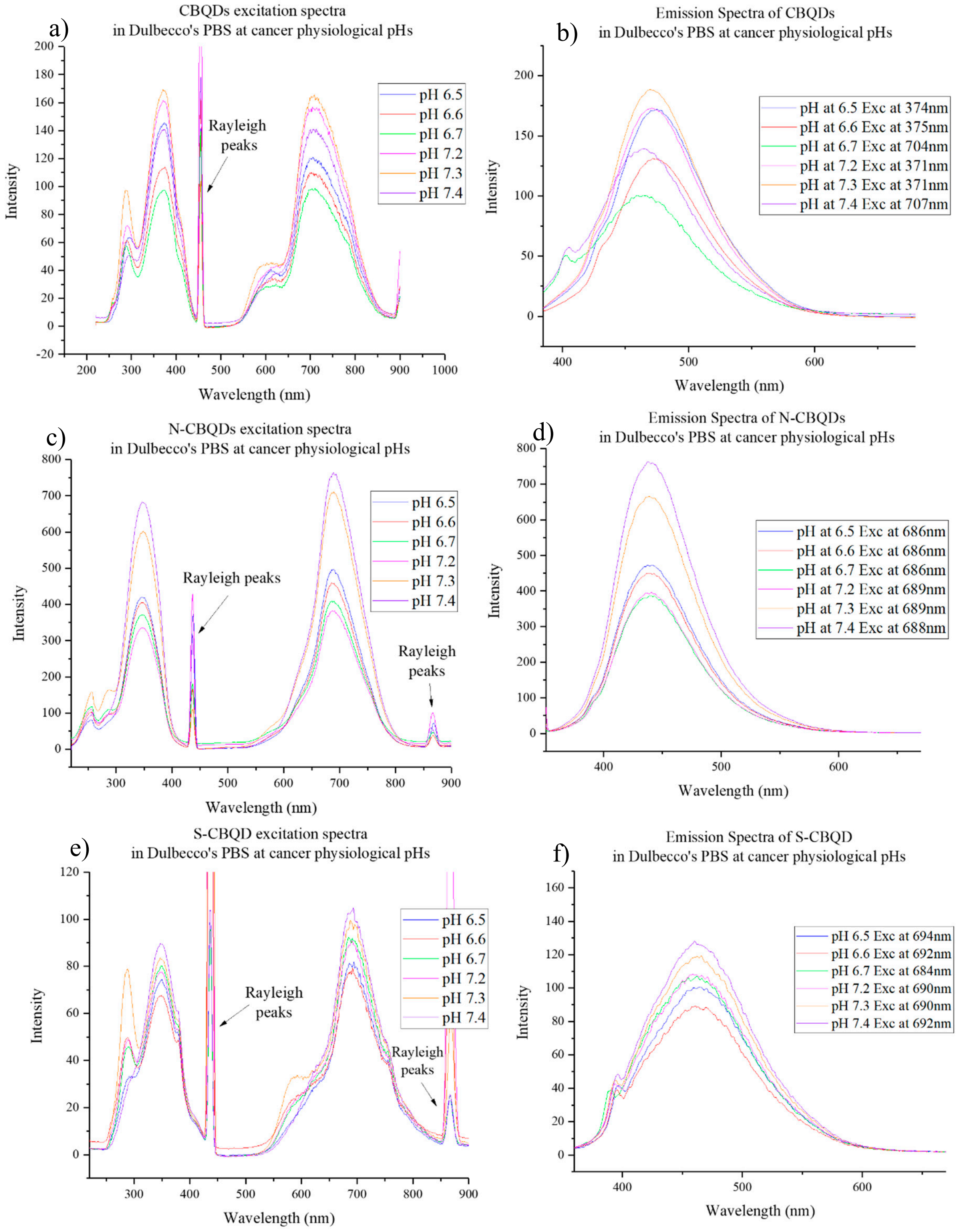

Figure S12: Excitation and emission spectra at different known physiological pHs present in cancer of a) CBQDs, b) CBQD-ADG, c) N-CBQD, d) NCBQD-ADG, e) S-CBQD, f) SCBQD-ADG.

Confocal Microscopy

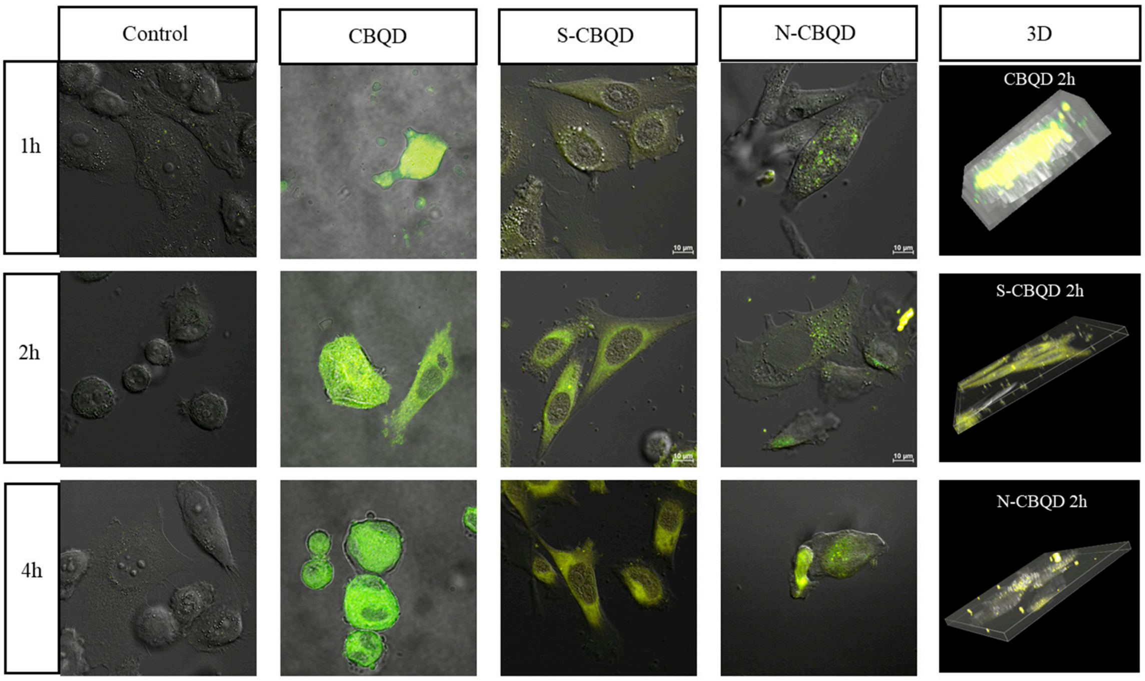

Figure S13: Confocal microscopy of PC-3 cells incubated with CBQD, S-CBQD and N-CBQD at different timeframes. Deconvolution was done via NIS-Elements Offline Deconvolution software. 3D images confirm material internalization.

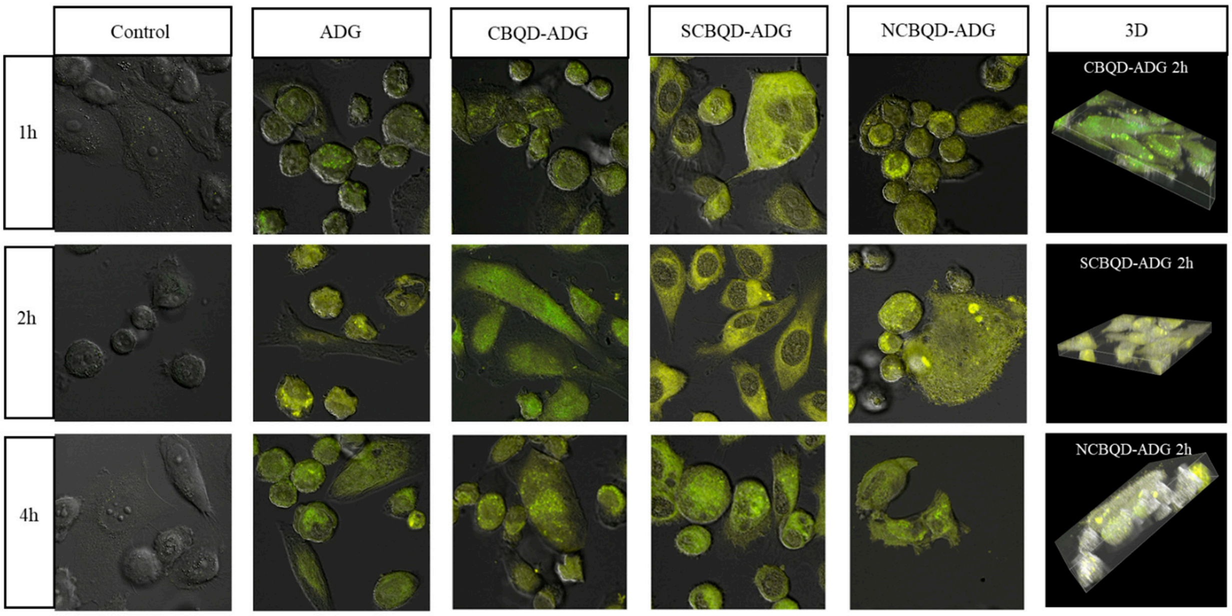

Figure S14: Confocal microscopy of PC-3 cells incubated with CBQD-ADG, SCBQD-ADG and NCBQD-ADG at different timeframes. Deconvolution was done via NIS-Elements Offline Deconvolution software. 3D images confirm material internalization.

Table S1: Confocal Microscopy Observations

	1 h incubation	2 h incubation	4 h incubation
ADG	Every area in the cell has fluorescence. Spheres with fluorescence may be observed around the cytoplasm and mainly in the nuclei.	Every area in the cell has fluorescence. Spheres with fluroescence may be observed around the cytoplasm and mainly in the nuclei.	Every area in the cell has fluorescence. Spheres's presence is less pronounced.
CBQD	Cytosol has fluorescence. No fluorescence in nuclei.	Cytosol has fluorescence. No fluorescence in nuclei.	Mitosis observed. Cytosol has fluorescence. No fluorescence in nuclei.
N-CBQD	Spheres with fluorescence are observed in the cytoplasm and in some cells a few spheres with fluorescence in the nuclei.	Spheres with fluorescence are localized near the nuclei, possibly Golgi apparatus or endoplasmic reticulum.	Spheres with fluorescence are localized near the nuclei, possibly Golgi apparatus or endoplasmic reticulum.

S-CBQD	Cytosol has fluorescence and around 2-3 spheres with fluorescence are observed per cell. No fluorescence in nuclei.	Cytosol has fluorescence and every cell has 1-2 spheres localized near the nuclei.	Some fluorescence entered the nuclei but most remains on the cytosol. No fluorescent spheres observed
CBQD-ADG	Many spheres with fluorescence observed throughout the whole cell.	Spheres with fluorescence observed throughout the whole cell but a bit less than at 1 h.	Increase in the number of spheres with fluorescence throughout the whole cell.
NCBQD-ADG	Spheres with fluorescence observed throughout the whole cell.	Spheres with fluorescence observed throughout the whole cell in comparable number as 1 h	Much less spheres with fluorescence observed throughout the whole cell and fluorescence is more dispersed throughout the cell.
SCBQD-ADG	Cytosol has fluorescence. No fluorescence in nuclei. Same observations as lone S-CBQD.	Spheres with fluorescence are observed now at 2 h. Some entry of fluorescence is observed in nucleolus but not the nucleus.	No entry to the nucleus, only to the nucleolus. Spheres with fluorescence are observed throughout the cytosol.
AuAgCBQD	Fluorescence is noticeably localized around the nucleus and less on the cytosol. Slight entry of fluorescence into nucleus.	Fluorescence is noticeably localized around the nucleus and less on the cytosol. Slight entry of fluorescence into nucleus. More spheres with fluorescence are observed around the cytosol.	Fluorescence is noticeably localized around the nucleus and less on the cytosol. Localization of spheres with fluorescence in a specific organelle on one side of the vicinity of the nucleus.
AuAgNCBQD	Spheres with fluorescence are biased in localization in one side of the cell. Consistent in all present cells. Cytosol has fluorescence.	Spheres with fluorescence are localized in one side of the cell and with higher intensity than at 1 h. Cytosol has fluorescence. Fluorescence entered the nucleolus but not the nuclei.	Fluorescence entered the nuclei in addition to the nucleolus. Less spheres with fluorescence observed.
AuAgSCBQD	Fluorescence observed in the cytosol. No fluorescence observed in nucleus. Some spheres	Fluorescence observed in the cytosol. Fluorescence observed in the nucleolus but not in nucleus. Some	Fluorescence observed in the cytosol. Fluorescence observed in the nucleolus but not in nucleus. Some

	with fluorescence observed.	spheres with fluorescence observed.	spheres with fluorescence observed.
AuAgCBQD-ADG	Fluorescence observed in the cytosol. Fluorescence observed in the nucleolus but not in nucleus. A few spheres with fluorescence observed.	Fluorescence observed in the cytosol. Fluorescence observed in the nucleolus but not in nucleus. A few spheres with fluorescence observed.	Fluorescence observed in the cytosol. Fluorescence observed in the nucleolus but not in nucleus. No few spheres with fluorescence observed.
AuAgNCBQD-ADG	Some fluorescence spheres observed around the nucleus, fluorescence observed in the cytosol localized notably in one side of the nucleus. No entry to nucleus. Entry to nucleolus.	Fluorescence observed in the cytosol. Many fluorescence spheres observed in the cytosol. No entry to nucleus. Entry to nucleolus.	Fluorescence observed in the cytosol. Many fluorescence spheres are observed in the cytosol. A bit of entry to nucleus. A lot of entry to nucleolus.
AuAgSCBQD-ADG	Some fluorescence spheres in the cytosol. Fluorescence observed in the cytosol. A bit of entry to nucleus. A lot of entry to nucleolus.	Some fluorescence spheres in the cytosol. Fluorescence observed in the cytosol. A bit of entry to nucleus. A lot of entry to nucleolus.	Fewer fluorescence spheres in the cytosol. Fluorescence observed in the cytosol. A bit of entry to nucleus. A lot of entry to nucleolus.

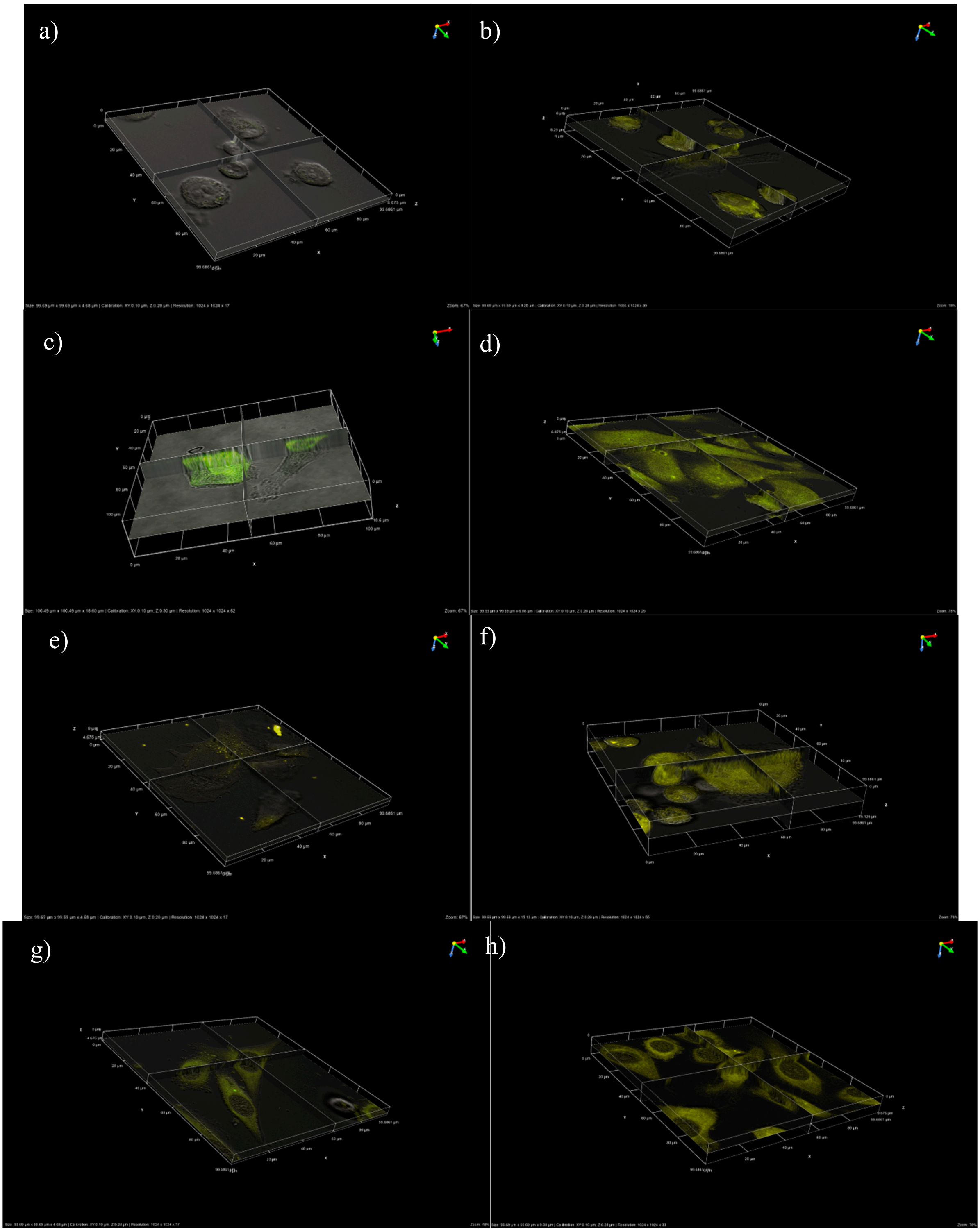
a)
b)
c)
d)
e)
f)
g)
h)

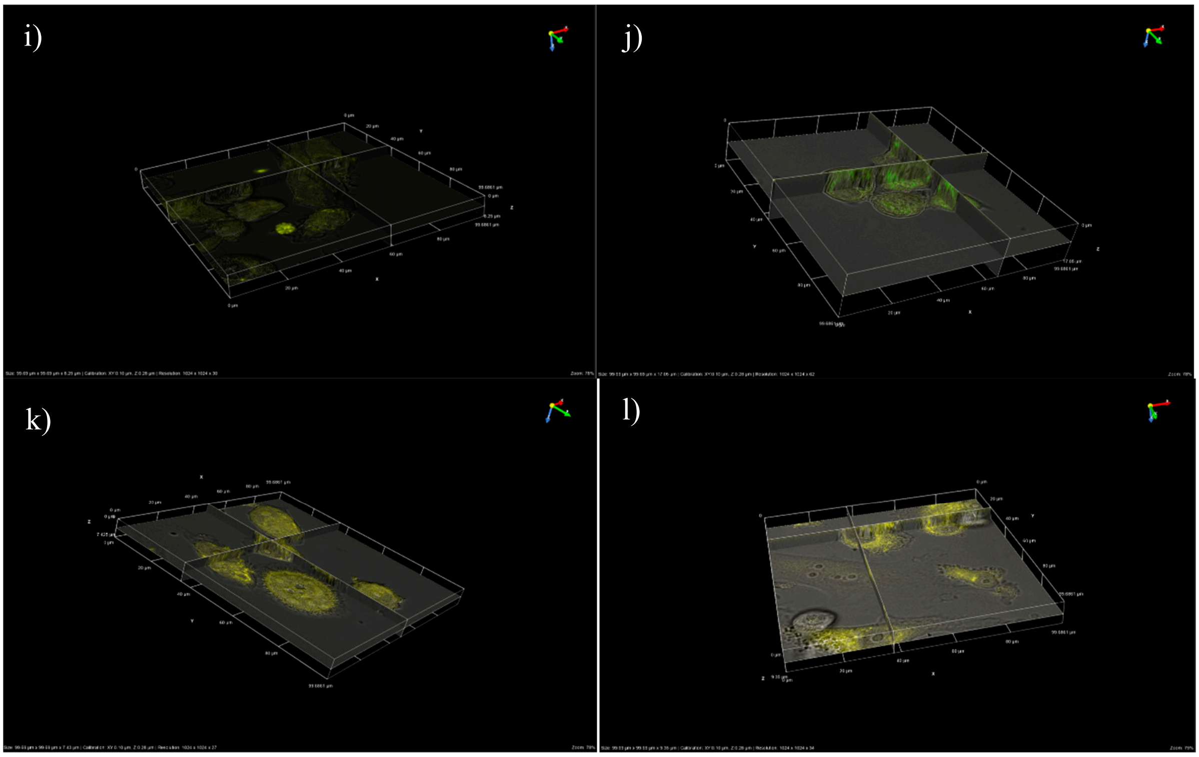

Figure S15: Virtual sectioning of 3D images. PC-3 cells treated with materials for 2 h. a) Control, b) ADG, c) CBQD, d) CBQD-ADG, e) N-CBQD, f) NCBQD-ADG, g) S-CBQD, h) SCBQD-ADG, i) AuAgCBQD, j) AuAgCBQD-ADG, k) AuAgNCBQD & l) AuAgNCBQD-ADG.

Surface enhanced Raman Spectroscopy (SERS)

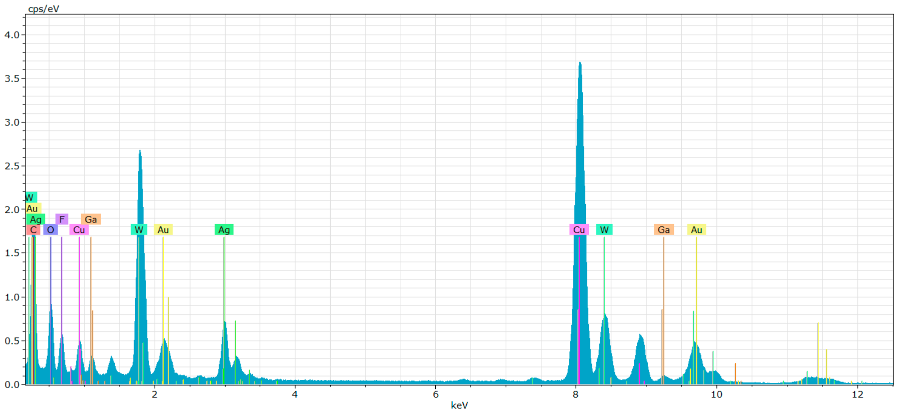

Figure S16: *In-situ* EDS spectra of Ag sputtered hydrophilic PVDF membrane. The presence of gold (Au) and tungsten (W) is due to FIB milling sample preparation for charge reduction.

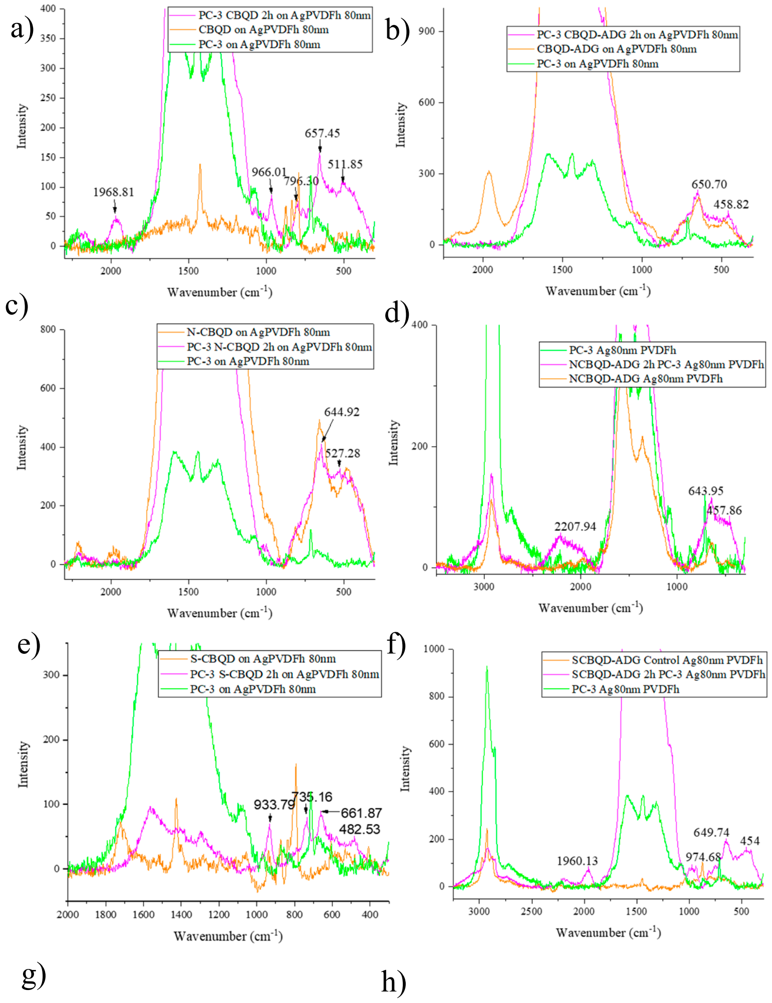

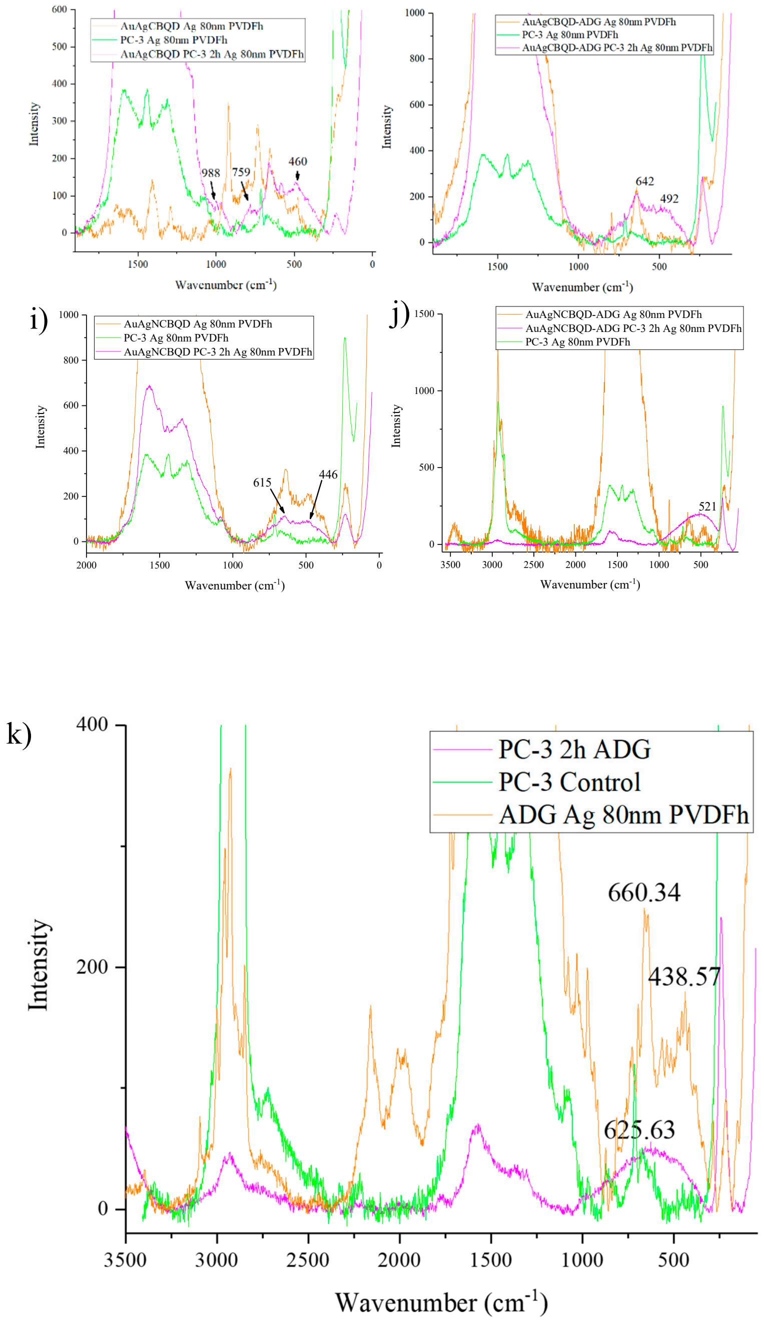

Figure S17: SERS spectra of the PC-3 cells incubated for 2 h with a) CBQDs, b) CBQD-ADG, c) N-CBQDs, d) NCBQD-ADG, e) S-CBQD, f) SCBQD-ADG, g) AuAgCBQDs, h) AuAgCBQD-ADG, i) AuAgNCBQD, j) AuAgNCBQD-ADG & k) ADG.

Table S2: Summary of SERS peaks within the PC-3 cells after 2 h incubation.

Material	Peak	Mode	Peak	Mode	Peak	Mode	Peak	Mode	Peak	Mode
CBQD	1968.81 cm^{-1}	Longitudinal optic (LO) + Acoustic (LA)	966.01 cm^{-1}	=C-H out of plane	796.30 cm^{-1}	H-C-C=C	657.45 cm^{-1}	C-C aliphatic	511.85 cm^{-1}	COO^{-} rocking
CBQD-ADG	650.70 cm^{-1}	C-C aliphatic	458.82 cm^{-1}	$-CH_2OH$						
S-CBQD	933.79 cm^{-1}	υ(C-O-C)	735.16 cm^{-1}	Asymmetric C-S-C	661.87 cm^{-1}	C-S stretch	482.53 cm^{-1}	C-O deformation		
SCBQD-ADG	974.68 cm^{-1}	(Unsat) $-CH=CH_2$	649.74 cm^{-1}	Ring deformation	454 cm^{-1}	$-CH_2OH$				
N-CBQD	644.92 cm^{-1}	δC-C-N	527.28 cm^{-1}	C-O-C Deform						
NCBQD-ADG	643.95 cm^{-1}	δC-C-N	457.86 cm^{-1}	$-CH_2OH$						
AuAg CBQD	988 cm^{-1}	=C-H out of plane	759 cm^{-1}	H-C-C=C	460 cm^{-1}	C-O-C				
AuAg CBQD-ADG	642 cm^{-1}	Ring deformation	492 cm^{-1}	C-O in-plane deformation						

AuAg SCBQD	529 cm^{-1}	δO-C-S	430 cm^{-1}	δC-H		
AuAg SCBQD -ADG	657 cm^{-1}	Ring deformation	569 cm^{-1}	in plane ring deformation	507 cm^{-1}	-CH-OH, C-O deformation
AuAg NCBQD	615 cm^{-1}	δC-C-N	446 cm^{-1}	C-O-C deform		
AuAg NCBQD -ADG	521 cm^{-1}	in plane ring deformation				

ANOVA results for MTS assay data sets

For all tests, alpha was set to 0.05.

Table S3: Anova Single Factor for AuAgCBQDs in PC-3 cells

SUMMARY

Groups	*Count*	*Sum*	*Average*	*Variance*
AuAgCBQD P1	9	327.5963	36.39959	764.0791
AuAgCBQD P2	9	312.5347	34.72607	568.4206
AuAgCBQD P3	9	353.8311	39.31457	626.8902

ANOVA

Source of Variation	*SS*	*df*	*MS*	*F*	*P-value*	*F crit*
Between Groups	97.05608	2	48.52804	0.074301	0.928605	3.402826
Within Groups	15675.12	24	653.13			

Total	15772.18	26				

Table S4: Anova: Single Factor for AuAgNCBQDs in PC-3 cells

SUMMARY

Groups	*Count*	*Sum*	*Average*	*Variance*
AuAgNCBQD P1	9	227.1244	25.23604	482.1681
AuAgNCBQD P2	9	278.4713	30.94125	238.1693
AuAgNCBQD P3	9	308.1833	34.24258	207.1312

ANOVA

Source of Variation	*SS*	*df*	*MS*	*F*	*P-value*	*F crit*
Between Groups	373.6983	2	186.8492	0.604384	0.554518	3.402826
Within Groups	7419.749	24	309.1562			
Total	7793.448	26				

Table S5: Anova Single Factor for AuAgSCBQDs in PC-3 cells

SUMMARY

Groups	*Count*	*Sum*	*Average*	*Variance*
AuAgSCBQD P1	9	300.5771	33.39745	1055.389
AuAgSCBQD P2	9	294.4868	32.72076	1012.691
AuAgSCBQD P3	9	373.1008	41.45565	792.6724

ANOVA

Source of Variation	*SS*	*df*	*MS*	*F*	*P-value*	*F crit*
Between Groups	425.0719	2	212.5359	0.222881	0.801848	3.402826
Within Groups	22886.02	24	953.5843			

Total	23311.1	26				

Table S6: ANOVA Single Factor for CBQDs in PC-3 cells

SUMMARY

Groups	*Count*	*Sum*	*Average*	*Variance*
Column 1	9	332.9277	36.99197	1028.275
Column 2	9	394.3142	43.81268	958.9777
Column 3	9	452.5226	50.28029	761.1826

ANOVA

Source of Variation	*SS*	*df*	*MS*	*F*	*P-value*	*F crit*
Between Groups	794.7951	2	397.3975	0.433771	0.653041	3.402826
Within Groups	21987.49	24	916.1452			
Total	22782.28	26				

Table S7: ANOVA Single Factor for N-CBQDs in PC-3 cells

SUMMARY

Groups	*Count*	*Sum*	*Average*	*Variance*
Column 1	9	338.3806	37.59785	356.3873
Column 2	9	372.3516	41.3724	579.8926
Column 3	9	461.0216	51.22462	453.3194

ANOVA

Source of Variation	*SS*	*df*	*MS*	*F*	*P-value*	*F crit*
Between Groups	891.0067	2	445.5033	0.961795	0.396454	3.402826
Within Groups	11116.79	24	463.1998			
Total	12007.8	26				

Table S8: ANOVA Single Factor for S-CBQDs in PC-3 cells

SUMMARY

Groups	*Count*	*Sum*	*Average*	*Variance*
Column 1	9	324.7148	36.07943	1193.669
Column 2	9	339.0555	37.67283	568.4632
Column 3	9	349.4181	38.82424	630.4999

ANOVA

Source of Variation	*SS*	*df*	*MS*	*F*	*P-value*	*F crit*
Between Groups	34.19595	2	17.09797	0.021438	0.978809	3.402826
Within Groups	19141.05	24	797.5439			
Total	19175.25	26				

Table S9: ANOVA Single Factor for CBQDs in RWPE-1 cells.

SUMMARY

Groups	*Count*	*Sum*	*Average*	*Variance*
Column 1	9	438.7841	48.75379	488.5499
Column 2	9	448.1216	49.79129	390.0128
Column 3	9	440.1364	48.90405	373.6378

ANOVA

Source of Variation	*SS*	*df*	*MS*	*F*	*P-value*	*F crit*
Between Groups	5.658472	2	2.829236	0.006778	0.993247	3.402826
Within Groups	10017.6	24	417.4001			
Total	10023.26	26				

Table S10: ANOVA Single Factor for N-CBQDs in RWPE-1 cells.

SUMMARY

Groups	*Count*	*Sum*	*Average*	*Variance*
Column 1	9	331.9619	36.88466	178.8542
Column 2	9	345.0631	38.34035	104.7127
Column 3	9	347.2317	38.5813	143.2481

ANOVA

Source of Variation	*SS*	*df*	*MS*	*F*	*P-value*	*F crit*
Between Groups	15.16706	2	7.583528	0.053303	0.948204	3.402826
Within Groups	3414.52	24	142.2717			
Total	3429.687	26				

Table S11: ANOVA Single Factor for S-CBQDs in RWPE-1 cells.

SUMMARY

Groups	*Count*	*Sum*	*Average*	*Variance*
Column 1	9	370.2364	41.13737	878.0149
Column 2	9	343.967	38.21855	710.4292
Column 3	9	377.3862	41.9318	806.7366

ANOVA

Source of Variation	*SS*	*df*	*MS*	*F*	*P-value*	*F crit*
Between Groups	68.81658	2	34.40829	0.043097	0.957893	3.402826
Within Groups	19161.45	24	798.3936			
Total	19230.26	26				
